# Elucidating Design Rules toward Enhanced Solid-State Charge Transport in Oligoether-Functionalized Dioxythiophene-Based Alternating Copolymers

**DOI:** 10.1021/acsami.3c00053

**Published:** 2023-07-14

**Authors:** Abigail A. Advincula, Amalie Atassi, Shawn A. Gregory, Karl J. Thorley, James F. Ponder, Guillaume Freychet, Austin L. Jones, Gregory M. Su, Shannon K. Yee, John R. Reynolds

**Affiliations:** † School of Materials Science and Engineering, Georgia Institute of Technology, Atlanta, Georgia 30332, United States; ‡ Materials and Manufacturing Directorate, Air Force Research Laboratory, Wright-Patterson Air Force Base, Ohio 45433, United States; § ARCTOS Technology Solutions, Dayton, Ohio 45432, United States; ∥ Center for Applied Energy Research, University of Kentucky, Lexington, Kentucky 40511, United States; ⊥ George W. Woodruff School of Mechanical Engineering, Georgia Institute of Technology, Atlanta, Georgia 30332, United States; # UES, Inc., Dayton, Ohio 45432, United States; ∇ NSLS-II, Brookhaven National Laboratory, Upton, New York 11973, United States; ○ School of Chemistry and Biochemistry, Georgia Tech Polymer Network, Center for Organic Photonics and Electronics, Georgia Institute of Technology, Atlanta, Georgia 30332, United States; ◆ Advanced Light Source and Materials Sciences Division, Lawrence Berkeley National Laboratory, Berkeley, California 94720, United States

**Keywords:** solid-state electrical conductivity, oligoether side chains, dioxythiophene polymers, charge transport

## Abstract

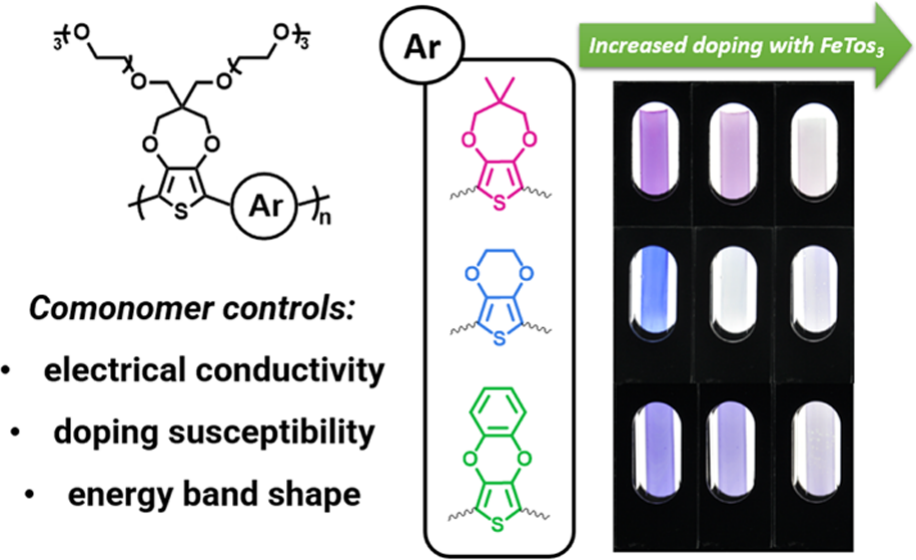

This study investigates the solid-state charge transport properties of the oxidized forms of dioxythiophene-based alternating copolymers consisting of an oligoether-functionalized 3,4-propylenedioxythiophene (ProDOT) copolymerized with different aryl groups, dimethyl ProDOT (DMP), 3,4-ethylenedioxythiophene (EDOT), and 3,4-phenylenedioxythiophene (PheDOT), respectively, to yield copolymers P(OE3)-D, P(OE3)-E, and P(OE3)-Ph. At a dopant concentration of 5 mM FeTos_3_, the electrical conductivities of these copolymers vary significantly (ranging between 9 and 195 S cm^–1^) with the EDOT copolymer, P(OE3)-E, achieving the highest electrical conductivity. UV–vis–NIR and X-ray spectroscopies show differences in both susceptibility to oxidative doping and extent of oxidation for the P(OE3) series, with P(OE3)-E being the most doped. Wide-angle X-ray scattering measurements indicate that P(OE3)-E generally demonstrates the lowest paracrystallinity values in the series, as well as relatively small π–π stacking distances. The significant (i.e., order of magnitude) increase in electrical conductivity of doped P(OE3)-E films versus doped P(OE3)-D or P(OE3)-Ph films can therefore be attributed to P(OE3)-E exhibiting both the highest carrier ratios in the P(OE3) series, along with good π–π overlap and local ordering (low paracrystallinity values). Furthermore, these trends in the extent of doping and paracrystallinity are consistent with the reduced Fermi energy level and transport function prefactor parameters calculated using the semilocalized transport (SLoT) model. Observed differences in carrier ratios at the transport edge (*c*_t_) and reduced Fermi energies [η(*c*)] suggest a broader electronic band (better overlap and more delocalization) for the EDOT-incorporating P(OE3)-E polymer relative to P(OE3)-D and P(OE3)-Ph. Ultimately, we rationalize improvements in electrical conductivity due to microstructural and doping enhancements caused by EDOT incorporation, a structure–property relationship worth considering in the future design of highly electrically conductive systems.

## 1 Introduction

Understanding solid-state charge transport in chemically doped, electrically conductive conjugated polymers (CPs) is important to emerging applications which include organic thermoelectrics,^1−4^ transparent electrodes,^5,6^ and bioelectronics.^7−9^ The synthetic tunability of CPs, e.g., backbone or side chain modulation, affords high control over energy levels (i.e., ionization energy/electron affinity), doping processes, and microstructures, factors that influence the electrical conductivity (σ) of a given CP system.^10−13^ Toward the goal of developing stable, highly electrically conductive films, structure–property relationships of 3,4-alkylenedioxythiophene (XDOT)-based polymers have been broadly investigated.^5,14−16^ Relative to their thiophene-based polymer analogues, XDOT-based polymers generally exhibit elevated degrees of doping (i.e., number of charge carriers per repeat unit)^13,17^ and decreased rates of de-doping (i.e., greater doping stability)^18^ due to their low ionization energies. Additionally, oligoether (OE) side chains have been increasingly employed with CPs (including XDOT-based materials) because of their ability to: (1) improve doping stability of CPs through enhanced polymer-ion miscibility,^19−23^ (2) lower CP bandgaps, and (3) improve charge transport through close π–π stacking distances (relative to their alkyl chain counterparts).^24,25^

While many XDOT-based units have been investigated for the development of stable, highly electrically conductive films, none have been as influential or as successful as 3,4-ethylenedioxythiophene (EDOT).^18,26^ The EDOT-based homopolymer serves as a primary component of poly(3,4-ethylenedioxythiophene):poly(styrenesulfonate) (PEDOT:PSS), a commercially available dispersion broadly used in research ranging from organic solar cells, to electrochromic and bioelectronic devices.^27−31^ PEDOT(OH), an EDOT-containing polymer derived from a soluble precursor, has recently been reported to demonstrate high, metal-like conductivity (>1000 S cm^–1^) as well as potentially useful stability (90% retention of σ after 2 months) under ambient conditions.^32^ Finally, a dioxythiophene copolymer composed of 2,2′-bis-(3,4-ethylenedioxy)thiophene (biEDOT) and 3,4-propylenedioxythiophene (ProDOT) substituted with branched oligoether side chains, PE_2_-biOE2OE3, exhibits an exceptionally high σ value for an OE-functionalized CP (∼400 S cm^–1^),^15^ a value 3–10× higher than comparable OE-functionalized thiophene-based analogues.^33−35^

By comparison, homopolymers composed of alternate XDOT units [e.g., ProDOT, 3,4-phenylenedioxythiophene (PheDOT), 3,6-dialkoxy-thieno[3,2-*b*]thiophene (DOTT)] yield lower σ values. P(ProDOT-EG), an OE-functionalized ProDOT-based homopolymer, was reported to have a σ value of 1 S cm^–1^ with the dopant F_4_TCNQ.^36^ Similarly, a PheDOT-based polymer (chemically polymerized with FeCl_3_) was reported to have a σ value of 1 S cm^–1^.^37^ The homopolymer of DOTT (HD) displayed σ values <10^–2^ S cm^–1^ when doped with either Magic Blue or F_4_TCNQ.^38^ Incorporation of unsubstituted EDOT into copolymer structures with monomers containing solubilizing groups, however, has been shown to increase the σ values of resultant oxidatively doped materials.^5,16,38^ While this EDOT incorporation is known to lower oxidation onsets by increasing the relative electron richness of copolymers relative to homopolymers (e.g., poly-ProDOT),^5,39^ it is not well known to what extent EDOT incorporation dictates the relationships between solid-state electrical conductivity, electronic structure, and microstructural properties in these conjugated copolymers.

**Figure 1:**
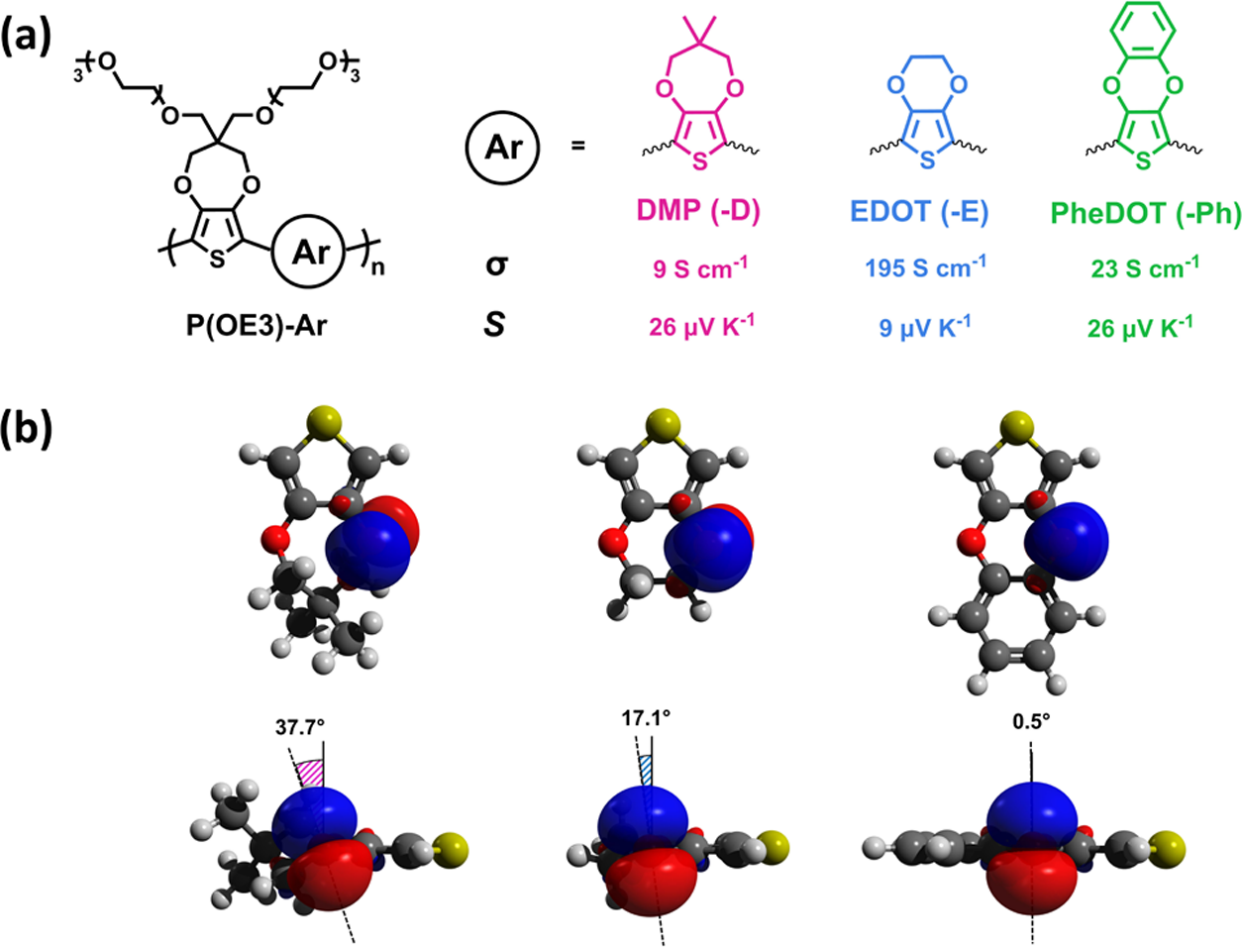
(a) Structures of the P(OE3)-D, P(OE3)-E, and P(OE3)-Ph with conductivity values (σ) and Seebeck coefficients (*S*) measured at a doping concentration of 5 mM FeTos_3_ in acetonitrile (ACN). (b) Top-view and side-view visualization of XDOT units (left to right: DMP, EDOT, and PheDOT) illustrate differences in XDOT unit planarity and oxygen lone-pair p-orbital orientations.^39^ Figure is reproduced with permission from ref (39).

**Figure 2:**
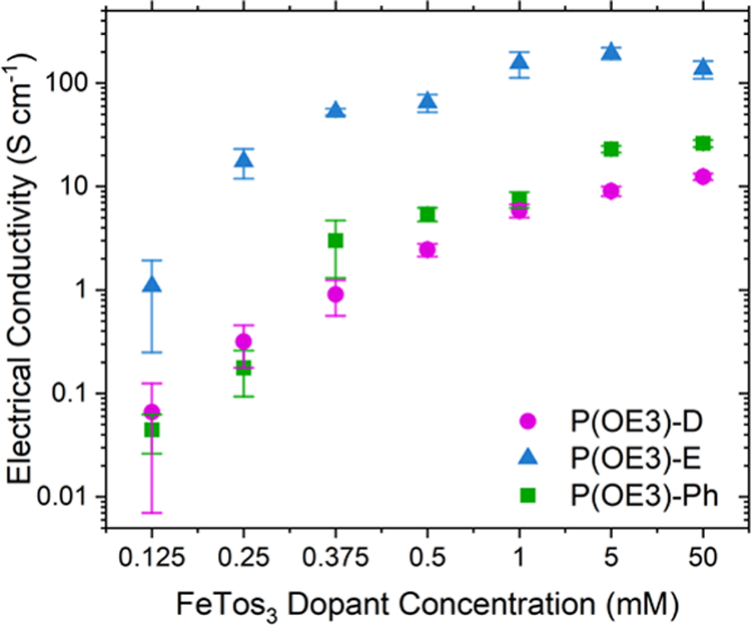
Measured electrical conductivities of blade-coated P(OE3) films doped with FeTos_3_ on glass substrates.

Herein, we study the solid-state charge transport properties of the oxidized forms of XDOT-based alternating copolymers consisting of an oligoether-functionalized ProDOT, P(OE3), copolymerized with different aryl (Ar) units: dimethyl ProDOT (DMP) (-D), EDOT (-E), and PheDOT (-Ph) (shown in Figure 1a). This work builds off our recent redox-focused study on the P(OE3)-Ar series, in which the fundamental electrochemical properties and applications were surveyed.^39^ In the current solid-state study, we elucidate how differences in XDOT comonomer planarity and resonance stabilization energy give rise to significant differences in thermoelectric properties of the P(OE3) series polymers in their oxidatively doped forms [σ from ∼10 to ∼200 S cm^–1^ with corresponding Seebeck coefficients (*S*) from +26 to +9 μV K^–1^ when doped with 5 mM ferric tosylate hexahydrate (FeTos_3_)]. To rationalize these differences in thermoelectric properties, spectroscopic and microstructural characterizations are used in conjunction with the semilocalized transport (SLoT) model,^17^ which accounts for varying localized (i.e., hopping-like) and delocalized (i.e., band-like) contributions to the observable transport properties. Doping processes are first probed by UV–vis–NIR spectroscopy, electrochemistry (i.e., cyclic voltammetry, differential pulse voltammetry, spectroelectrochemistry), and X-ray photoelectron spectroscopy (XPS). EDOT-containing P(OE3)-E demonstrates the greatest susceptibility to oxidative doping and yields the highest carrier ratios (ratio of charges per aromatic ring) of the P(OE3) series. Doping processes can furthermore be related to the resonance stabilization energies of the comonomer oxygen lone pairs with respect to: (1) the tilt of the oxygen p-orbital relative to the thiophene ring [DMP (37.7°), EDOT (17.1°), and PheDOT (0.5°), as shown in Figure 1b], and (2) the presence of competing sites for orbital delocalization, as robustly discussed in our redox-focused P(OE3) study.^39^ Grazing incidence wide-angle X-ray scattering (GIWAXS) is used to analyze the ordering of pristine and FeTos_3_-doped P(OE3) films, with P(OE3)-E demonstrating small π–π stacking distances and relatively low paracrystallinity. Finally, the SLoT model is used to quantify key charge transport parameters, i.e., the reduced Fermi energy and charge transport prefactor, to contextualize the high σ values obtained for P(OE3)-E compared to other polymers in the series. Ultimately, this work shows how subtle changes to backbone structure can give rise to large variations in solid-state properties, informing the design of future high-performing CP systems.

## 2 Experimental Section

### 2.1 Materials

Synthesis for the P(OE3) series by a C-H activation polymerization has been previously reported.^39^ The number-average molecular weights and dispersities of the polymers are 27 kg mol^–1^ and *Đ* = 2.9 for P(OE3)-D, 13 kg mol^–1^ and *Đ* = 2.0 for P(OE3)-E, and 20 kg mol^–1^ and *Đ* = 4.2 for P(OE3)-Ph (as measured by gel permeation chromatography vs polystyrene standards in CHCl_3_ at 40 °C).

Details on additional reagents and dopants can be found in the Supporting Information.

### 2.2 Sample Preparation

Polymer films were blade-coated from either chloroform/chlorobenzene or chloroform solutions. Specific solution concentrations, blade heights, and speeds were optimized for each polymer, with full details listed in the Supporting Information.

The films were doped by drop-casting FeTos_3_/acetonitrile (ACN) solutions of varying molarities (0.125–50 mM), followed by washing with clean ACN and drying. Full experimental details outlining dopant solution preparation and doping time can be found in the Supporting Information.

### 2.3 Electrical Conductivity and Seebeck Coefficient Measurements

Electrical measurements were performed using the four-probe van der Pauw technique, with film thicknesses determined by profilometry following the measurements. For Seebeck coefficient measurements, films were suspended between temperature-controlled Peltier stages and the voltage difference between contacts over each stage was measured over a series of temperature differences, with the Seebeck coefficient being extracted from the resulting *V* vs Δ*T* slope. Additional details for transport measurements can be found in the Supporting Information.

### 2.4 Optical Measurements

UV–vis–NIR results were obtained on thin polymer films on glass that were blade-coated to an optical density of 1.1 ± 0.05. Films were then doped with FeTos_3_ in a manner comparable to that performed for transport measurements, as detailed in the Supporting Information.

For spectroelectrochemistry, films were spray-coated to an optical density of 1.1 ± 0.05 onto ITO/glass substrates. Prior to the spectroelectrochemical measurements, polymer films were electrochemically conditioned (see the Supporting Information for additional details) in 0.1 M 1-ethyl-3-methylimidazolium tosylate (EMITos)/propylene carbonate (PC).

### 2.5 Electrochemistry

Electrochemical experiments were performed under inert atmosphere with 0.1 M EMITos/PC serving as the electrolyte, a silver wire pseudo-reference electrode as the reference electrode, a Pt flag as a counter electrode, and a glassy carbon button electrode as the working electrode in a three-electrode cell. Polymer films were prepared by drop-casting from chloroform onto the glassy carbon electrodes, with additional details outlined in the Supporting Information.^43^

### 2.6 XPS Measurements

XPS spectroscopy was performed on films cast and doped identical to those used for transport measurements. Peak deconvolutions were performed using Thermo Avantage analysis software, with additional instrumentation and analysis details in the Supporting Information.^40^

### 2.7 GIWAXS Measurements

GIWAXS measurements were performed at Brookhaven National Lab at the 12-ID Soft Matter Interfaces (SMI) beamline of the National Synchrotron Light Source II (NSLS-II). The polymer films were prepared by blade-coating onto silicon wafers and doping in the same manner comparable to those described for transport measurements. Further details of the analysis of the GIWAXS measurements are included in the Supporting Information.^41^

### 2.8 DFT Calculations

All density functional theory (DFT) calculations were run using Gaussian 16 Rev A.03^42^ and NBO 6.0,^44^ with the polymers modeled as 16 repeating thiophene units and geometries optimized in the gas phase using ωB97XD/6-31G*.^45^ Full computational details can be found in the Supporting Information.

## 3 Results and Discussion

### 3.1 Initial Conductivity Measurements of the P(OE3) series

The σ values of FeTos_3_-doped P(OE3) films, prepared via blade-coating, were measured using a four-point van der Pauw method. We note the measured electrical conductivities are approximately the same in all directions, and that blade-coating has not produced chain alignment in a specific direction. Table S1 tabulates the measured film thicknesses after doping. As the samples measured are relatively thick (>200 nm), we believe that the measured electrical conductivities are representative of bulk properties; consequently, we do not expect to see differences in electrical conductivity based on film thickness. Furthermore, due to the strength of the chosen dopant (FeTos_3_) to oxidize dioxythiophene-based polymers, exposure time of the P(OE3) films to the dopant solution is limited to 1 min for the electrical conductivity measurements, as well as for the subsequent spectroscopic (i.e., UV–vis–NIR and XPS) measurements (further details in the Supporting Information).

In Figure 2 and Table S2, σ values of the P(OE3) series show a marked increase when doped with increasing FeTos_3_ dopant concentrations. In general, P(OE3)-E values are one to two orders of magnitude higher than P(OE3)-D and P(OE3)-Ph, reaching maximum values at higher dopant concentrations (*ca*. 0.50 mM and above). Furthermore, temperature-dependent electrical conductivity measurements from 150 to 300 K show that the most electrically conductive copolymer, P(OE3)-E, is also the least thermally activated (Figure S1). Normalized temperature-dependent σ data (σ/σ_*T* = 300 K_), for blade-coated P(OE3) films doped with 5 mM FeTos_3_, demonstrates a shallower slope for the P(OE3)-E system relative to P(OE3)-D and P(OE3)-Ph. To investigate how these differences in σ arise, we first turn to a spectroscopic analysis of the polymers at varying doping levels.

### 3.2 Spectroscopic Analysis of the P(OE3) Series

**Figure 3:**
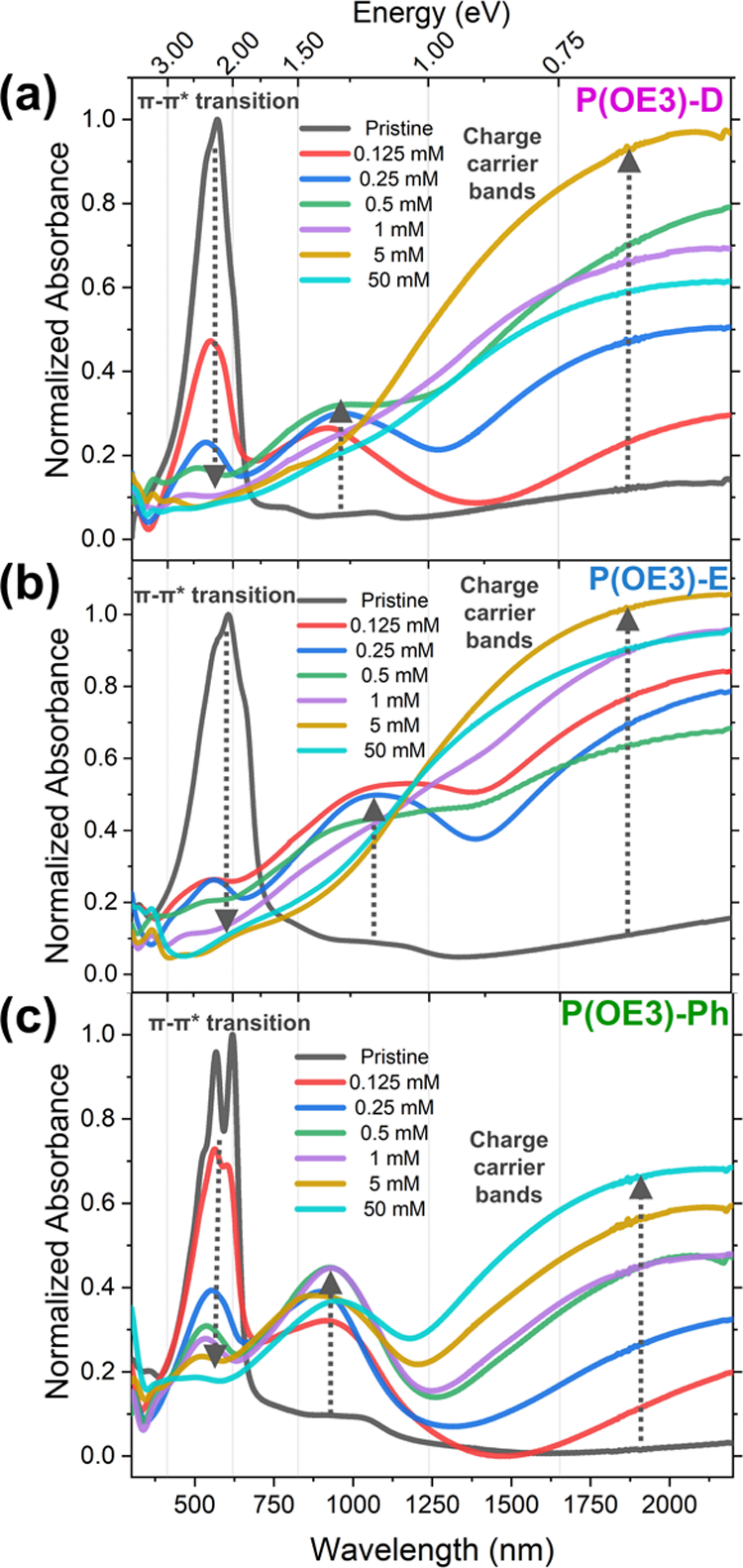
Composite UV–vis–NIR spectra for (a) P(OE3)-D, (b) P(OE3)-E, (c) P(OE3)-Ph films doped with different concentrations of FeTos_3_/ACN for 1 min. Note: as the as-cast blade-coated P(OE3)-E films demonstrated significant oxidation in air (consistent with our previous study^39^), P(OE3)-E films were treated with hydrazine vapors to achieve a similarly reduced state to the as-cast blade-coated P(OE3)-D and P(OE3)-Ph films, prior to being chemically doped with FeTos_3_.

Susceptibility to oxidative doping is first probed spectroscopically using UV–vis–NIR spectroscopy. Films on glass were blade-coated for thin-film UV–vis–NIR to an optical density of 1.1 ± 0.05 and doped with FeTos_3_/ACN solutions immediately prior to optical measurement. The extent of doping manifests as a decrease of the π–π* transition and a concomitant appearance of charge carrier bands in the near-IR. Photography of doped blade-coated films shows complete color bleaching for P(OE3)-E at 0.125 mM FeTos_3_ and more gradual bleaching for P(OE3)-D and P(OE3)-Ph (Figure S2). This is further exhibited in the UV–vis–NIR spectra of these films (shown here in Figure 3 as composite images of each polymer set), where the π–π* transition is bleached at different dopant concentrations (Figure 3). At the lowest dopant concentration (0.125 mM FeTos_3_), the π–π* transition of P(OE3)-E is nearly fully depleted while the same transition is only partially depleted for P(OE3)-D and P(OE3)-Ph; total bleachings at low dopant concentrations have been similarly observed for other EDOT-containing polymers.^46^ To achieve comparable bleaching to P(OE3)-E doped with 0.125 mM FeTos_3_, P(OE3)-D and P(OE3)-Ph must be doped with 0.25 and 1 mM FeTos_3_ solutions, respectively. Ultimately, the high susceptibility to oxidative doping of P(OE3)-E compared to that of P(OE3)-D or P(OE3)-Ph can be related to the electron richness of the P(OE3)-E polymer, as demonstrated experimentally and computationally in our prior redox-focused study on the P(OE3) series.^39^ Trends in oxidation onsets as measured by differential pulse voltammetry (DPV) (Figure S3) and bleaching onsets as measured by spectroelectrochemistry (Figure S4) corroborate these results (see the Additional Discussion on Electrochemistry and Spectroelectrochemistry in the Supporting Information). Previously reported cyclic voltammograms show each polymer to have a broad redox response with onsets consistent with this current work’s DPV results (albeit not perfectly matching due to the different electrolyte used), as well as redox stability to at least 1000 cycles.^39^

The evolution of the charge carrier bands in the near-IR, as demonstrated by differences in peak location and intensity, is similarly dependent on the comonomer selected and the dopant solution concentration. In the higher-energy charge carrier peaks developed between 700 and 1375 nm (Figure 3), the peak maxima is observed at different wavelengths and doping concentrations: P(OE3)-E (λ_max_ = 1070 nm at 0.125 mM FeTos_3_), P(OE3)-D (λ_max_ = 930 nm at 0.5 mM), and P(OE3)-Ph (λ_max_ = 900 nm at 1 mM). Additionally, at higher doping concentrations (i.e., 5 and 50 mM), full bleaching of this higher-energy charge carrier band is observed for P(OE3)-E and P(OE3)-D but not P(OE3)-Ph, consistent with the spectroelectrochemistry data (Figure S4). As an in-depth examination of charge carrier Coulombic interactions complicates the simple assignment of a singular charge carrier species (e.g., polarons or bipolarons) to distinct optical ranges,^47^ we consider further speculation of the evolving nature of the charge carrier (as it relates to changes in optical spectra) to be beyond the scope of this report. However, differences in both the bleaching of the π–π* transition and the development of charge carriers in the near-IR indicate a strong effect of comonomer selection on the susceptibility to oxidative doping of the resultant polymer, a point further explored in XPS measurements of the P(OE3) series.

The average carrier ratios (*c*, charges per aromatic ring) of the doped P(OE3) copolymer films were measured by XPS (see Figures S5–S8, Tables S3 and S4, and Additional Discussion on XPS Analysis). We note that films used for XPS and films used for electrical conductivity measurements come from the same batch of blade-coated films, with the only difference being that the films used for electrical conductivity measurements have contacts deposited on them prior to doping. Films are then doped immediately prior to their respective measurements (further details in the Supporting Information). As measurements have been done across multiple samples, we assert that the error bars accurately capture the sample-to-sample variation and that this gives us highly comparable films across the XPS and electrical conductivity measurements.

**Figure 4:**
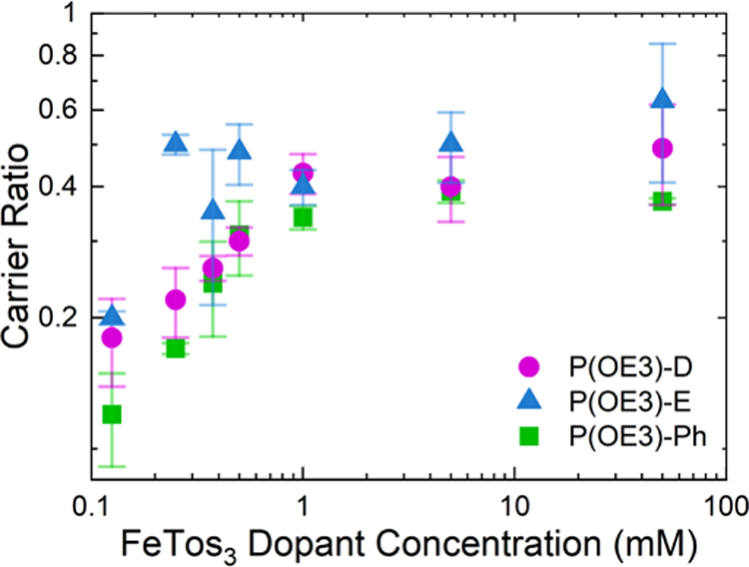
Charge carrier ratio as a function of FeTos_3_ dopant concentration for films of the P(OE3) copolymer series.

Figure 4 plots *c* as a function of FeTos_3_ concentration, and Table S5 tabulates these *c* values with additional information on the approximate number of carriers per thiophene ring. Overall, the carrier ratios are highest for P(OE3)-E, followed by P(OE3)-D and P(OE3)-Ph, confirming the general trends in susceptibility to oxidative doping observed in both the solid-state and potential-dependent UV–vis–NIR spectra. At lower FeTos_3_ dopant concentrations (0.25–0.50 mM), P(OE3)-E reaches ∼50% higher carrier ratios (*c* = 0.35–0.50) than P(OE3)-D (*c* = 0.22–0.30) and P(OE3)-Ph (*c* = 0.17–0.31). At higher dopant concentrations (e.g., 5 and 50 mM FeTos_3_), P(OE3)-E only reaches ∼20% higher carrier ratios (*c* = 0.50–0.63) than P(OE3)-D (*c* = 0.40–0.49) or P(OE3)-Ph (*c* = 0.39–0.37). To contextualize these values, P3HT exhibits a maximum *c* of around 0.33,^48^ while several PEDOT studies have reported *c* of nearly 0.5,^49−51^ comparable to the calculated values for this polymer series.

While *c* trends with σ values for the P(OE3) copolymer series, the percentage differences in *c* do not scale with the order of magnitude differences in σ. At a dopant concentration of 5 mM FeTos_3_, for example, P(OE3)-D and P(OE3)-E have *c* of 0.40 and 0.50, respectively, while the σ corresponding to these films are 9 and 195 S cm^–1^. This discrepancy in the scaling of charge carrier ratios and electrical conductivity values suggests different charge carrier mobility and other fundamental transport parameters.^52^ To further probe differences in transport, theoretical energy levels and delocalization of charge carriers on model hexadecamer systems were calculated using DFT.

### 3.3 DFT Calculations

Theoretical energy levels and charge carrier delocalizations for the P(OE3) copolymer series were investigated using a DFT-tuned range-separated functional approach (ωB97XD/6-31G*). To first determine the smallest model system to be fully representative of a polymer (as opposed to an oligomer), a model PEDOT system with 24 repeat units was investigated. EDOT was chosen as the unit for this test system, as its lack of side group atoms (either the methyl or appending phenylene ring) made the calculation less computationally expensive. Charge distributions were first calculated for the 24-mer in both the neutral and oxidized states. The differences between these two states were then used to calculate the Hirshfeld charge per fragment shown in Figure S9, which visualizes the delocalization of the charge carrier species over the polymer (each fragment represents a repeat unit “ring”). The Hirshfeld charges span fragments 7 through 22 (shaded in gray), which corresponds to the charge carrier delocalizing over 16 rings. Therefore, we believe a hexadecamer (16-mer) to be the smallest system fully representative of a polymer rather than an oligomer. Utilization of 16-mer systems in this current work serves as a significant improvement upon our prior usage of 12-mer systems for modeling the P(OE3) series,^39^ as using a larger model system allows us to place higher confidence in the validity of the DFT calculations.

To model the P(OE3) series of polymers, DFT calculations were performed on model hexadecamers (the model for P(OE3)-E is shown in Figure S10a as an example). It should be noted that the OE chain has been converted to methyl groups for computational accessibility. Isosurfaces of the highest occupied molecular orbitals (HOMOs) for each system (shown in Figure S10b) show that the HOMO lies predominantly over the middle rings; rings at either end of the model hexadecamer (i.e., rings 1 and 16) contribute <1% to the total charge. This finding confirms our assertion that a model hexadecamer is large enough to allow a clear measure of the charge carrier delocalization. Calculated theoretical ionization energy (IE) values (shown in Table S6) are both consistent with prior theoretical IE calculations^39^ and found to be in good agreement with the measured onsets of oxidation (obtained from Figure S3), with P(OE3)-E requiring the least amount of energy to remove an electron, followed by P(OE3)-D and P(OE3)-Ph. These trends can be understood in the context of oxygen lone-pair resonance stabilization energies calculated from NBO analysis of the DMP, EDOT, and PheDOT units, as discussed in depth in our previous redox-focused study.^39^

Charge carrier delocalizations were investigated using the model hexadecamer systems. Increased charge carrier delocalization contributes to decreased charge carrier binding energy (i.e., coupling between the electronic and geometric structures), thereby maximizing charge transport.^53^ Hirshfeld charge analysis provides a quantitative description of molecular charge distribution (delocalization) for different atomic fragments and has been previously used to model polymer charge carrier species.^54−58^ Charge distributions were first calculated for the hexadecamers in both the neutral and oxidized states. The differences between these two states were then used to calculate the Hirshfeld charge per fragment shown in Figure S11, which visualizes the delocalization of the charge carrier species. Summation of the Hirshfeld charge per fragment (Table S7) for P(OE3)-E and P(OE3)-D reveals that charge is delocalized equally over P(OE3) and the respective comonomers: [50% P(OE3), 50% EDOT] and [50% P(OE3), 50% DMP]. Summations for P(OE3)-Ph reveal similar charge delocalizations over P(OE3) and PheDOT [49% P(OE3), 51% PheDOT]. Significant charge carrier localization on a particular unit, as demonstrated for copolymers with donor-acceptor design motifs,^54,57^ is therefore not observed for the P(OE3) copolymer series. This can be attributed to all of the units being XDOT-based and therefore not as strongly contrasting in electron-donating or electron-accepting character from each other. Additionally, high planarity between rings on the polymer chain (i.e., dihedral angles close to 180°) is observed for the P(OE3) series in both the neutral and oxidized states (Figure S12), consistent with the literature.^59−61^ The lack of twisting along the backbone for any of the polymers therefore contributes to the strong charge carrier delocalization observed for all polymers.

In conclusion, observed redox trends are consistent with IE values calculated by DFT, indicating that differences in electron donation into the polymer backbone, as robustly discussed in our previous study,^39^ do play a role in the subtle variation of the oxidation potentials by DPV and the more drastic differences in polymer thin-film bleaching by solid-state UV–vis–NIR. DFT can therefore rationalize the development of higher charge carrier ratios at lower oxidation potentials/doping concentrations for P(OE3)-E due to this polymer’s low IE relative to P(OE3)-D and P(OE3)-Ph. However, Hirshfeld charge carrier per fragment calculations seem to indicate that developed charges delocalize to the same extent over the polymer chain. This similarity across the polymer series suggests that differences in solid-state microstructure (e.g., π–π overlap and local ordering) are likely to be a greater factor in determining electrical conductivity than the electronic properties calculated for a single polymer chain, which is limited in representing the energetic landscape of an oxidatively doped film. To elucidate differences in measured electrical conductivity, we therefore turn to microstructural analysis of these materials.

### 3.4 Microstructural Analysis of P(OE3) Series

Thermogravimetric analysis (TGA) and differential scanning calorimetry (DSC) were first employed to probe changes to thermal transitions associated with changes to molecular structure. Each material displayed thermal stability up to 325 °C under an inert argon atmosphere by TGA (Figure S13a). The lack of any distinct thermal features in the DSC traces (i.e., glass-transition or melting peaks) indicates no significant microstructural changes occurring when heating or cooling the samples (Figure S13b), consistent with other OE-functionalized CP systems.^54,58^ Local ordering observed in the GIWAXS of the pristine P(OE3) films (*vide infra*) is therefore ascribed to aggregation in the polymer solution prior to processing the film, a point supported by the similarity of the solid-state UV–vis–NIR in Figure 3 and the solution UV–vis of the P(OE3) series published in our previous redox-focused study.^39^

**Figure 5:**
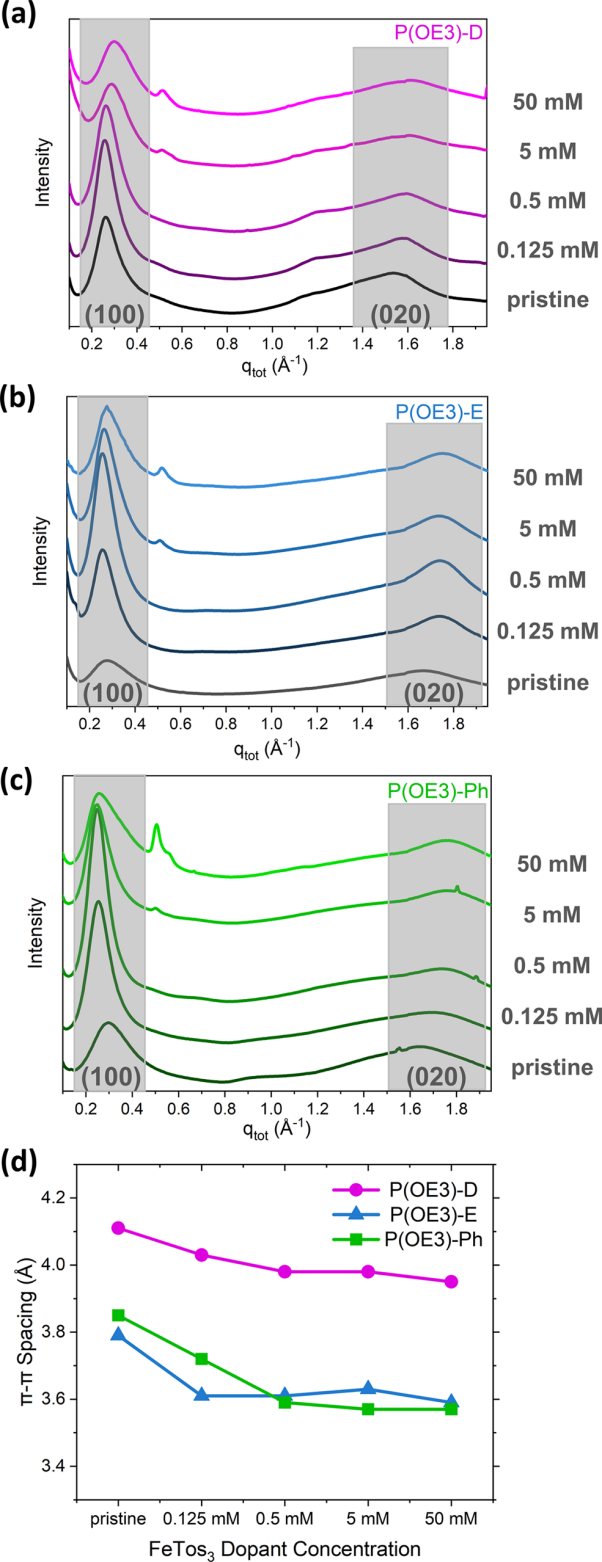
Radially integrated GIWAXS profiles of pristine films and 0.125, 0.5, 5, and 50 mM FeTos_3_-doped films of (a) P(OE3)-D, (b) P(OE3)-E, and (c) P(OE3)-Ph. Shaded areas show regions of the (100) and (020) features. (d) π–π spacings calculated from the GIWAXS profiles as a function of dopant concentration.

To provide insight into the structure of these materials, GIWAXS analysis was performed on pristine and doped films. Figures S14–S16 show the two-dimensional GIWAXS diffractograms of pristine films and films doped at varying FeTos_3_ concentrations. Figure 5a–c shows the radially integrated GIWAXS profiles of the P(OE3) series, with the regions associated with lamellar stacking (100) and π–π stacking (020) shaded. Figures S17 and 5d show lamellar and π–π spacings as a function of dopant concentration, respectively.

We first consider the lamellar spacings (100) of the P(OE3) series shown in Figure S17 (tabulated in Table S6). In the pristine state, the P(OE3)-D lamellar spacing (24.0 Å) is larger than that of either P(OE3)-E (22.4 Å) or P(OE3)-Ph (21.2 Å). Upon doping at 0.125 mM FeTos_3_, we observe an initial increase in lamellar stacking distances for P(OE3)-E (an increase of 2.1 Å) and P(OE3)-Ph (an increase of 3.6 Å), consistent with dopant insertion into the side chains.^12,62^ At higher dopant concentrations, we observe diverging trends in the lamellar spacings. From the 0.125 mM doped film to the 50 mM doped film, the lamellar spacing of P(OE3)-D decreases by 3.5 Å and P(OE3)-E decreases by 2.1 Å. Over the same range of dopant concentrations, however, P(OE3)-Ph maintains lamellar spacings close to 25 Å. Simultaneously, we note the emergence of additional features at ∼0.5 Å^–1^ for the P(OE3) series at dopant concentrations of 5 and 50 mM, with the features of P(OE3)-Ph at this position being especially prominent (Figure 5a–c). We note that these features at ∼0.5 Å^–1^ are likely not from higher-order lamellar reflectance, as the ∼0.5 Å^–1^ features are not consistently located at an integer spacing with respect to the (100) peak. Instead, these features are attributed to the formation of ordered dopant domains, akin to what is observed in some F4TCNQ-doped films.^12^

We next consider the π–π spacings (020) of the P(OE3) series shown in Figure 5d (values tabulated in Table S8). In the pristine state, the π–π spacings of P(OE3)-E (3.79 Å) and P(OE3)-Ph (3.85 Å) are smaller than the P(OE3)-D π–π spacing (4.11 Å), consistent with the high unit planarity of EDOT and PheDOT relative to DMP (previously shown in Figure 1).^39^ The π–π spacings for each of the polymers decrease upon doping up to 50 mM FeTos_3_, decreasing by 0.16 Å for P(OE3)-D, 0.20 Å for P(OE3)-E, and 0.28 Å for P(OE3)-Ph. At the different doping levels, however, closer π–π spacings are maintained for P(OE3)-E and P(OE3)-Ph relative to P(OE3)-D. As decreased π–π spacings lead to increased molecular overlap as well as carrier delocalization and mobility between chains,^12^ the observed trends are in good agreement with electrical conductivity trends observed in Figure 2.

Finally, we evaluate the paracrystalline disorder parameter (*g*), which represents the statistical fluctuation in *d*-spacing (in this case π–π spacing) in the crystallite regions of a polymer which can prevent long-range order.^63^ Statistical fluctuation in crystalline order affects transport and charge-trapping properties of conducting and semiconducting materials,^64^ and increased crystallite ordering (low *g* values) is positively correlated with polaron delocalization, higher mobilities, and increased σ.^62,65^ Paracrystallinity values, associated with intermolecular π–π stackings for pristine and doped films of polymers in the P(OE3) copolymer series, were calculated using a pseudo-Voigt peak fit (example fit in Figure S18) and tabulated in Table S8. While the P(OE3) films, both pristine and doped, do not exhibit significant long-range order (*g* ≥ 10%),^63,64^ trends may be observed for the different polymers at different dopant concentrations (see Figure S19). For P(OE3)-D, *g* values hover between 15 and 16% for both pristine and doped films, indicating that molecular doping has relatively little effect on the statistical fluctuation of the π–π spacings. The *g* values for P(OE3)-E, in contrast, decrease from ∼16 to ∼12% from a pristine to a 0.125 mM FeTos_3_ doped film, indicating an ordering effect in the crystallite regions upon doping. The *g* values for P(OE3)-Ph also decrease from ∼19% for the pristine film to ca. 13–14% for the doped films, suggesting a similar ordering effect to that observed in the P(OE3)-E system. Ultimately, the low overall *g* values of the P(OE3)-E films (both pristine and doped) indicate lower statistical fluctuation of π–π spacings and a higher degree of crystallite ordering for P(OE3)-E relative to P(OE3)-D and P(OE3)-Ph. Increased order of the P(OE3)-E system further contributes to the higher σ values observed for this system relative to P(OE3)-D and P(OE3)-Ph.^62,65^

### 3.5 Effect of Comonomer Selection on P(OE3) Series Transport Parameters, Analyzed through the SLoT Model

Finally, the SLoT model is used to evaluate the charge transport properties of the P(OE3) copolymers doped with FeTos_3_. Up to this point, differences in σ values in the P(OE3) polymer series have been understood in the context of susceptibility to oxidative doping, carrier ratio, π–π spacings, and paracrystallinity values. P(OE3)-E has the highest electrical conductivities of the P(OE3) series, likely because it obtains the highest carrier ratios, close π–π spacings, and the lowest paracrystallinity upon doping. Under similar doping conditions, P(OE3)-D and P(OE3)-Ph tend toward similar and lower electrical conductivity values due to their lower carrier ratios, larger π–π spacings, and higher paracrystallinity values. While these chemical and structural changes are sufficient to explain the higher σ values observed for P(OE3)-E relative to P(OE3)-D and P(OE3)-Ph, further analysis of temperature-dependent Seebeck coefficients (*S*), electrical conductivities (σ), and carrier ratios (*c*) through the SLoT model allows for differences in charge transport parameters (electronic band shape, localization energy, etc.) to be calculated and contextualized.^17^

The SLoT model takes the form^17^
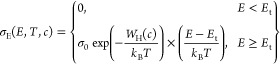
1

where σ_E_ (*E*, *T*, *c*) is the charge transport function, and the charge transport function is a function of the charge carrier energy (*E*), temperature (*T*), and carrier ratio (*c*). A full description of the SLoT model can be found in refs (13, 16, 17). The SLoT charge transport function is then related to the measurable thermoelectric (σ, *S*) properties by evaluating the transport function using the Boltzmann transport equation, yielding
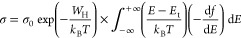
2and
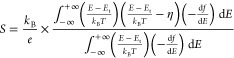
3

By fitting measured σ and *S* values to eqs 2 and 3, one can glean a deeper physical understanding of the differences in observable charge transport properties.

**Figure 6:**
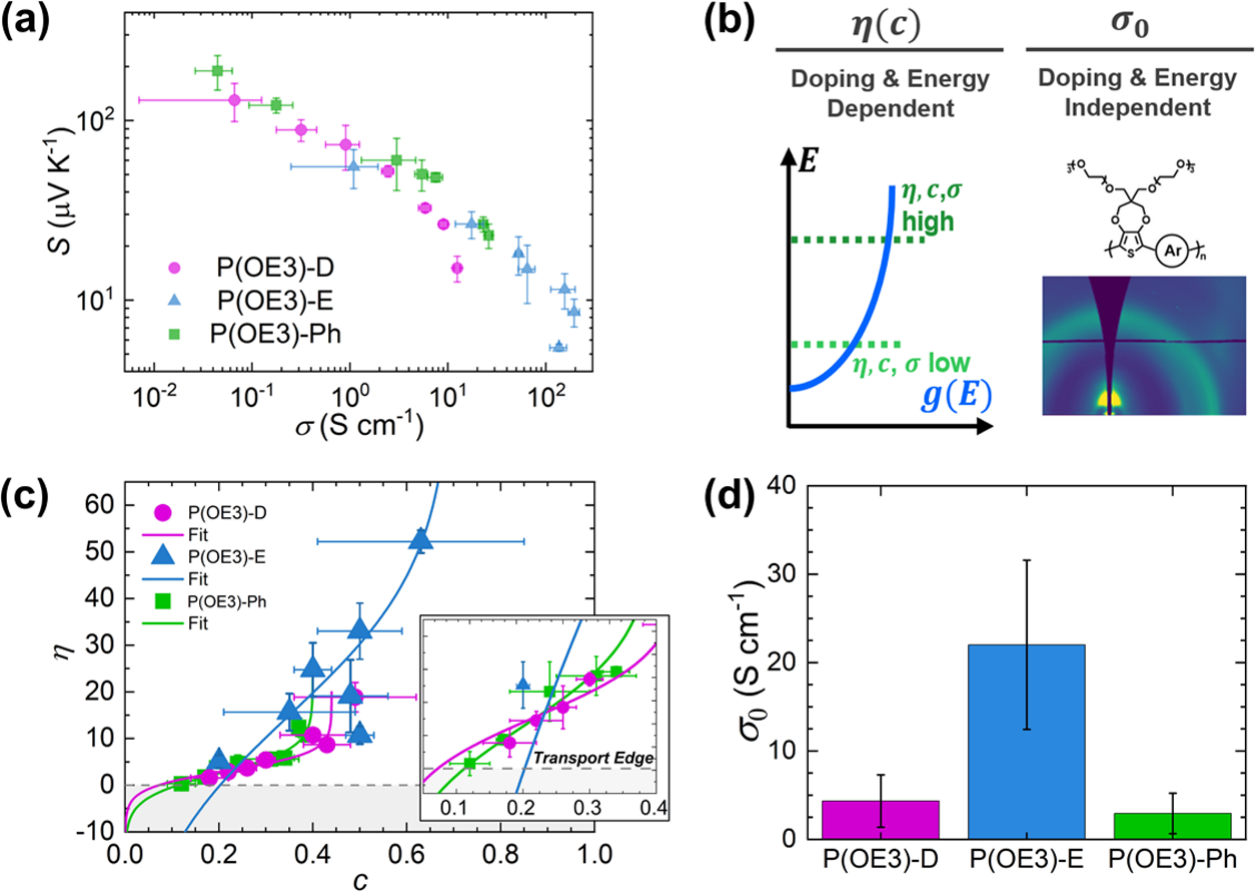
Charge transport analysis of the P(OE3) series. (a) Thermoelectric values of the P(OE3) series depicted with an *S–*σ plot. Points left to right indicate increasing doping concentrations with FeTos_3_ (0.125, 0.25, 0.375, 0.5, 1, 5, 50 mM). Error bars represent ± one standard deviation from at least three unique films at that doping level. (b) Graphical illustration of the SLoT transport parameters. η, the reduced Fermi energy level, is dependent on the density of electronic states, *g*(*E*), indicated by the blue curve. As *c* increases, η and σ increase, while absolute Seebeck, |*S*|, decreases. σ_0_, the doping- and energy-independent transport function prefactor, is suggested to be constant for a polymer-dopant-processing system. (c) SLoT model analysis of the reduced Fermi energy as a function of carrier ratio, η(*c*). The inset depicts the η(*c*) values near the transport edge, *E*_t_. (d) Average σ_0_ values for each polymer system. The nominal σ_0_ value represents the polymer average across multiple doping levels and films, and the error bars represent ± one standard deviation.

Figure 6a shows the measured thermoelectric values of FeTos_3_ doped films of the P(OE3) series. We observe the expected *S*–σ anticorrelation,^17^ showing that σ values increase and *S* values decrease with increasing FeTos_3_ doping concentrations; increasing FeTos_3_ doping concentrations increases the carrier ratio (*c*), as quantified using XPS (Figure 4). At lower doping levels (higher Seebeck coefficients), the P(OE3)-D, P(OE3)-E, and P(OE3)-Ph polymers have comparable curvatures, slopes, and nominal values in Figure 6a; however, at higher doping levels, P(OE3)-E obtains ∼10× higher σ values. To understand the difference in charge transport exhibited by P(OE3)-E compared to P(OE3)-D and P(OE3)-Ph, the charge transport parameters η(*c*) and σ_0_ are evaluated.

The high σ values exhibited by P(OE3)-E, relative to P(OE3)-D and P(OE3)-Ph, can be better contextualized by examining key charge transport expressions: the reduced Fermi energy as a function of carrier ratio  and the SLoT transport prefactor, σ_0_ (Figure 6b). Inferences about the electronic band (represented here by the density of electronic states function, *g*(*E)*) can be made using the reduced Fermi energy relationship, η(*c*), using eq 3; each material system has a unique η(*c*) relationship that depends on the electronic states allowed by the structure of the polymer.^16^ As *c* increases, charge carriers increasingly populate the electronic band, *g*(*E*), at higher and more mobile energy levels, thereby increasing η and σ. In contrast, the SLoT transport prefactor (σ_0_), calculated from eq 2, relates to constants such as the charge carrier mobility prefactor and effective mass of a charge carrier, which are independent of the energy of the system and the doping level. Lastly, while germane to the charge transport discussions of other XDOT-based systems,^13,16,17^ discussion on localization energy (*W*_H_) values for the P(OE3) series (shown in Figure S20) are left to the Additional Discussion in the SI as the differences in *W*_H_ are not sufficient to explain the differences in σ in this P(OE3) series. Ultimately, the SLoT model provides a quantitative framework for isolating these complex interrelationships by isolating η(*c*) and σ_0_ expressions.

First, we examine the η(*c*) relationship in Figure 6c, as this expression quantifies the position of the Fermi energy level with respect to the transport edge as a function of doping level (*c*). For every measured Seebeck coefficient in Figure 6a, eq 3 can be evaluated to calculate a single η value as a function of carrier ratio (values are listed in full in Table S9). Figure 6c shows that as the carrier ratio (*c*) increases, η increases, with each material system having a unique η(*c*) relationship, as indicated by the differences in curve slope and shape. The transport edge (*E*_t_) is denoted by the dashed horizontal line, and the carrier ratio needed to exceed the transport edge is referred to as *c*_t_ (the fit intercept at η = 0). The transport edge is oftentimes synonymous with the band edge and marks the onset of significant transport.^66^ Here, the *c*_t_ value is specific to the polymer: P(OE3)-D (*c*_t_ = 0.07), P(OE3)-E (*c*_t_ = 0.20), and P(OE3)-Ph (*c*_t_ = 0.11). For context, P(OE3)-D and P(OE3)-Ph have *c*_t_ values close to P3HT (*c*_t_ ∼ 0.05), while P(OE3)-E has *c*_t_ values more consistent with PEDOT (*c*_t_ ∼ 0.22).^17^ With regards to the η(*c*) relationship, P(OE3)-E achieves η values ranging from 30 to 50 at the highest doping levels while P(OE3)-D and P(OE3)-Ph achieve η values ranging from 10 to 20 at their highest doping levels. Physically, this signifies P(OE3)-E charge carriers accessing more mobile, higher-energy states than P(OE3)-D and P(OE3)-Ph charge carriers. The steeper η(*c*) curve for P(OE3)-E is further attributed to increased resonance stabilization of the EDOT unit vs DMP or PheDOT (as discussed previously in Figure 1b),^39^ and increasing η as a function of increasing EDOT fraction is consistent with previous studies on dioxythienothiophene copolymers.^14,16,38^ Ultimately, these observed differences in *c*_t_ values and maximum η values suggest a broader electronic band (better overlap and more delocalization)^67^ for the EDOT-incorporating P(OE3)-E polymer relative to P(OE3)-D and P(OE3)-Ph (see Figure S21). Density of states calculated for the hexadecamers using DFT (see Figure S22) appear to corroborate this claim, as the highest energy band for P(OE3)-E does appear to be a little broader relative to the highest energy bands for P(OE3)-D and P(OE3)-Ph. However, this analysis is complicated by the onset of the lower energy band centered around −7 eV for P(OE3)-Ph as well as likely differences in electronic structure between a gas-phase hexadecamer DFT calculation and a solid-state polymer film.

Second, the SLoT model additionally enables the calculation of σ_0_, the SLoT transportation prefactor, which is approximated to be a constant for a materials system. In inorganic semiconductors, σ_0_ is oftentimes related to inherent carrier mobilities and effective masses.^66^ Through the SLoT model, σ_0_ may be similarly used to quantify these transport properties for CPs, representing an “idealized” electrical conductivity independent of localization energy or reduced Fermi energy. Figure S23 shows that σ_0_ is independent to η, consistent with the SLoT model. Figure 6d shows the average σ_0_ for the P(OE3) series; notably, P(OE3)-Ph and P(OE3)-D have σ_0_ values that are ∼2 S cm^–1^ while P(OE3)-E has σ_0_ values that are ∼20 S cm^–1^. These significant differences indicate that P(OE3)-E has the potential to be 10× more conductive than P(OE3)-D and P(OE3)-Ph in the absence of localization effects and at equal η values. In Figure S24, we show that this difference in σ_0_ is consistent with calculated drift mobility (μ, calculated using XPS carrier densities) and weighted mobility (μ_*w*_, calculated using a linear transport function) values, which show that P(OE3)-E has mobilities an order of magnitude higher than P(OE3)-E and P(OE3)-Ph. Physically, we believe this difference in σ_0_ may be related to the combination of increased ordering (lower paracrystallinity values) and smaller π–π spacings in the crystalline domains of the P(OE3)-E doped films. Ultimately, this ∼10× increase in σ_0_ with a ∼2× increase in η is consistent with the 10–20× larger σ values observed for EDOT-incorporating P(OE3)-E relative to P(OE3)-D and P(OE3)-Ph.

## 4 Conclusions

In this work, we investigate design rules toward enhanced solid-state transport properties in OE-functionalized dioxythiophene-based alternating copolymers, providing a mechanistic understanding of observed increases in σ through spectroscopic, computational, and scattering measurements as well as further contextualization through the SLoT model. We observe that P(OE3)-D and P(OE3)-Ph exhibit similar electrical conductivities (i.e., within the same order of magnitude), while P(OE3)-E electrical conductivities are significantly higher at all doping concentrations. By XPS, doped P(OE3)-E films yield the highest charge carrier ratios in the series, followed by P(OE3)-D and then P(OE3)-Ph. P(OE3)-E’s susceptibility to oxidative doping is additionally supported by UV–vis–NIR spectroscopy (significant suppression of the π–π transition and growth of the charge carrier bands at low doping concentrations) and electrochemical measurements (lower onset of oxidation of P(OE3)-E relative to P(OE3)-D and P(OE3)-Ph). GIWAXS is further employed to probe differences in π–π overlap and local ordering (i.e., paracrystallinity). We note that P(OE3)-D demonstrates the largest π–π spacings (poorest π–π overlap) in the P(OE3) series, while P(OE3)-E and P(OE3)-Ph trend toward similar π–π spacings at the different doping concentrations. Oxidatively doped P(OE3)-D therefore exhibits higher charge carrier ratios, but poorer π–π overlap relative to P(OE3)-Ph, resulting in P(OE3)-D and P(OE3)-Ph yielding similar macroscopic electrical conductivities. In contrast, P(OE3)-E exhibits both the highest carrier ratios in the P(OE3) series as well as good π–π overlap and local ordering (low paracrystallinity values), elucidating the significant (i.e., order of magnitude) increase in electrical conductivity of doped P(OE3)-E films versus doped P(OE3)-D or P(OE3)-Ph films.

Finally, the SLoT model is used to further contextualize the observable transport properties (i.e., *S* and σ), with an ∼10× increase in σ_0_ and an ∼2× increase in η quantifying the 10–20× larger σ values observed for P(OE3)-E relative to P(OE3)-D and P(OE3)-Ph. EDOT incorporation ultimately enables the development of high charge carrier densities and local order in the resultant polymers, significantly broadening the electronic band structure of EDOT-containing polymers. Ultimately, this work quantifies our previously qualitative understanding of how EDOT incorporation significantly changes material properties, informing the design of future high-performing CP systems for solid-state thermoelectric and electronic properties.

## Supporting Information Available

Synthetic and instrumentation details as well as detailed experimental methods (PDF)
