# Supplementary material for: Elucidating Design Rules toward Enhanced Solid-State Charge Transport in Oligoether-Functionalized Dioxythiophene-Based Alternating Copolymers
Source: ACS Appl Mater Interfaces. 2023 Jul 14;15(29):35227–38. doi: 10.1021/acsami.3c00053 (PMC10375480; doi:10.1021/acsami.3c00053)
Supplement: Supplementary file 1 — am3c00053_si_001.pdf [file am3c00053_si_001.pdf]

Supporting Information:

Elucidating Design Rules Towards Enhanced Solid-State Charge Transport in Oligoether-Functionalized Dioxythiophene-Based Alternating Copolymers

Abigail A. Advincula,[a,b,c] Amalie Atassi,[a] Shawn A. Gregory,[a] Karl J. Thorley,[d] James F. Ponder Jr.,[b,e,f] Guillaume Freychet,[g] Austin L. Jones,[h] Gregory M. Su,[i] Shannon K. Yee,[e] and John R. Reynolds[a,h *]

[a]*School of Materials Science and Engineering, Georgia Institute of Technology, Atlanta, GA 30332 USA*

[b]*Air Force Research Laboratory, Materials and Manufacturing Directorate, Wright-Patterson Air Force Base, OH 45433 USA*

[c]*ARCTOS Technology Solutions, Dayton, OH 45432 USA*

[d]*Center for Applied Energy Research, University of Kentucky, Lexington, KY 40511 USA*

[e]*George W. Woodruff School of Mechanical Engineering, Georgia Institute of Technology, Atlanta, GA 30332 USA*

[f]*UES, Inc., Dayton, OH 45432 USA*

[g]*NSLS-II, Brookhaven National Laboratory, Upton, NY 11973 USA*

[h]*School of Chemistry and Biochemistry, Georgia Tech Polymer Network, Center for Organic Photonics and Electronics, Georgia Institute of Technology, Atlanta, GA 30332 USA*

[i]*Advanced Light Source & Materials Sciences Division, Lawrence Berkeley National Laboratory, Berkeley, CA 94720 USA*

*E-mail: reynolds@chemistry.gatech.edu

Table of Contents

Additional Materials 3
Sample Preparation 3
  Film Preparation 3
  Doping of Films for Transport and Spectroscopic Measurements 3
Instrumentation Details and Additional Discussion of Analysis: 4
  Electrical Conductivity and Seebeck Coefficient Measurements 4
  Optical Measurements 4
  Photography 5
  Electrochemistry 5
  XPS Measurements 5
  Thermal Measurements 5
  GIWAXS Measurements and Analysis 5
  DFT Calculations 6
Additional Discussions 7
  Electrochemistry 7
  Spectroelectrochemistry 7
  XPS Analysis 7
  The SLoT Model and Charge Transport Analysis of W_H 9
Additional Figures 11
Additional Tables 29
References 33

Additional Materials:

Ferric tosylate hexahydrate ($FeTos_3$, technical grade) and 1-ethyl-3-methylimidazolium tosylate (EMITos, ≥98%) were obtained from Sigma-Aldrich and used as received. Acetonitrile (ACN) was obtained from Fisher Scientific and dried in a solvent purification system to be used as the supporting electrolyte.

Sample Preparation:

Film Preparation. Blade-coated films were prepared using a Zehnter Testing Instruments blade coater (a ZAA 2300 quipped with a ZUA 2000 blade). All solutions were stirred overnight. For the P(OE3)-D films, a 15 mg/ml solution of P(OE3)-D in 1:1 chloroforom/chlorobenzene was used, with a blade speed of 40 mm/s and a blade height of 250 μm above the glass substrate height. For the P(OE3)-E films, a 20 mg/ml solution of P(OE3)-E in chloroform was used, with a blade speed of 40 mm/s and a blade height of 150 μm above the glass substrate height. For the P(OE3)-Ph films, a 20 mg/ml solution of P(OE3)-Ph in chloroform was used, with a blade speed of 40 mm/s and a blade height of 150 μm above the glass substrate height.

Doping of Films for Transport and Spectroscopic Measurements. Films of the P(OE3) series were prepared by blade-coating from either chloroform or chloroform/chlorobenzene solutions. The films were doped by drop-casting 0.1 mL of the ferric tosylate/ACN solutions of varying molarities (0.125 mM, 0.25 mM, 0.375 mM, 0.5 mM, 1 mM, 5 mM, 50 mM), allowing the film to saturate with the dopant solution.[1] From the moment of dopant solution deposition, a countdown timer for precisely 1 minute was started. Upon expiration of the timer, the films were then immediately rinsed in an excess of ACN (10 mL of ACN drop-cast on the film) and then dried in a fume hood for ~5 minutes. Measurements (transport or spectroscopic) were then taken on the same day. Additionally, we note that the dopant solutions were prepared via serial dilution to ensure accurate concentrations. Note: A 1 minute exposure time of the P(OE3) films to the dopant solution was chosen because it provided a long enough time to allow for total dopant penetration of the films but short enough such that the measured spectra and electrical conductivities were primarily influenced by dopant concentration, as opposed to extensive exposure to the dopant solution.

Instrumentation Details and Additional Discussion of Analysis:

Electrical Conductivity and Seebeck Coefficient Measurements. Electrical measurements were performed using an in-house setup. Tungsten tipped micromanipulators were used to make electrical contact to the platinum contact pads, and sheet resistance was acquired based on the four-probe Van der Pauw technique. From this, the electrical conductivity was obtained by correcting for film thickness, as determined by profilometry. For Seebeck coefficient measurements, substrate supported films were suspended between temperature-controlled Peltier stages (separated ~3 mm), and a series of temperature differences, up to $\Delta T = 10$ °C, were applied between the stages above and below the nominal Peltier stage temperature. The thermoelectric voltage was measured between two contact pads on separate stages using the probe tips, with the temperature of each side of the film being measured with a K- type thermocouple near the probe tips, while the voltage (V) and temperature data was acquired using a Keithley 2700 DMM with a 7708 Mux card using a LabVIEW interface. The Seebeck coefficient was extracted as the slope of the V vs. ΔT. To obtain temperature-dependent properties, the nominal Peltier stage temperature was changed from 20 – 70 °C using temperature controllers (Model LFI-3751). Measures were performed in at least triplicate on different films to capture sample-to-sample variation.

Optical Measurements. Thin-film UV-vis-NIR were obtained on an Agilent Cary 5000 spectrophotometer in 1 cm path length quartz cuvettes scanning from 300-2200 nm.

Films on glass were blade coated for thin-film UV-vis-NIR. Films were blade-coated to an optical density of 1.1 ± 0.05. (Note: P(OE3)-E films display significant air-doping/air-oxidation under ambient conditions as demonstrated in our previous study on the P(OE3) series.[2] To achieve a reduced state comparable to as-cast P(OE3)-D and P(OE3)-Ph films, the P(OE3)-E films were treated with hydrazine vapors.) Films were then chemically doped with $FeTos_3$ in the manner described in the section "Doping of Films for Transport and Spectroscopic Measurements." To obtain the normalized absorbances, absorbances taken of the undoped (or hydrazine reduced, as in the case of P(OE3)-E) films were normalized to an absorbance of 1. The values used to normalize the undoped films were then applied to the doped films.

Films on transparent electrode substrates were fabricated for spectroelectrochemistry. Films were spray-coated to an optical density of 1.1 ± 0.05 onto ITO/glass substrates (7 mm x 50 mm, sheet resistance 8-12 Ω sq^{-1}, Delta Technologies Ltd). Prior to the spectroelectrochemical

measurements, polymer films were electrochemically conditioned (cycled 10×) between -1.05 to +0.45 V vs. Fc/Fc^+ in 0.1 M EMITos/propylene carbonate (PC). Potential-dependent spectra were recorded using a Cary 5000 UV-Vis-NIR spectrophotometer.

Photography. Photography was performed using a Nikon D90 SLR camera with a Nikon 18-105 mm VR lens. No manipulation aside from cropping of the original image was applied.

Electrochemistry. All electrochemical experiments were performed under inert atmosphere and with thoroughly purged electrolyte systems The electrolyte used was 0.1 M EMITos/PC. For measurements in organic electrolyte, a silver wire pseudo-reference electrode ($E_{1/2}$ for ferrocene: 410 mV) was used as the reference electrode. The redox response of the films was characterized using differential pulse voltammetry (DPV), which was performed in a three-electrode cell using an EG&G Princeton Applied Research 273A potentiostat/galvanostat, under CorrWare control (step size 2 mV, step time 0.1 s, amplitude 5 mV). A glassy carbon working electrode (0.07 cm^2) was polished with 0.1 and 0.05 µm alumina powder and rinsed rigorously with water before use. Polymers were dissolved in 4 mg/ml in chloroform. To fabricate films on glassy carbon electrodes, 2 µL of this solution was drop-cast on the electrode surface and left to dry at room temperature.

XPS Measurements. XPS spectra were recorded on a Thermo K-Alpha instrument under ultra-high vacuum (< 10^{-7} mbar) using an Al Kα source (1.486 keV) equipped with a charge-neutralizing flood gun. High resolution, elemental scans were recorded at a 0.1 eV energy resolution and a pass energy of 50 eV. XPS peak deconvolution was performed using Thermo Avantage analysis software. Each of the analyses was performed with a Smart background: a Shirley background with the additional constraint that the background shall not exceed the intensity of the data collected. Symmetric line shapes were used with a convolution of Gaussian and Lorentzian functions, the relative proportion being 30% Lorentzian.[3]

Thermal Measurements. Thermogravimetric analysis (TGA) was performed on a Mettler Toledo TGA2 Start System. Samples were run under an inert atmosphere (argon) at a scan rate of 10 °C/minute from 30 °C to 600 °C. Differential scanning calorimetry (DSC) was performed on a TA Instruments DSC 250. Samples were run under an inert atmosphere (argon) at a heating and cooling rate of 10 °C/minute between -50 °C to 300 °C in hermetically sealed aluminum pans with pinholes.

GIWAXS Measurements and Analysis. GIWAXS measurements were performed at Brookhaven National Lab at the 12-ID Soft Matter Interfaces (SMI) beamline of the National

Synchrotron Light Source II (NSLS-II) with a beam energy of 14 keV. The polymer samples were prepared by blade-coating onto silicon wafers and doping procedures in the same manner as described for solid-state sample preparation. The 2D scattering patterns were collected at an X-ray incidence angle of 0.15° with a Pilatus 900 K−W detector with a pixel size of 172 μm placed at 279 mm from the sample. The sample and detector were enclosed in a vacuum chamber to suppress air scatter. To cover the range of scattering angles desired, the vertically oriented elongated detector was moved horizontally on a fixed arc and images were later visualized in Xi-CAM software[4] and stitched and radially integrated using custom code. The GIWAXS profiles (q_{tot}) are radially integrated values of the q-vectors (no constraint in terms of azimuthal range).

Peak positions in the lamellar regions were taken to be the q-value at the peak maximum. Peak positions ($d_{(020)}$) and full width half maximum (Δq) for the (020) peaks were obtained using the Analysis>Peaks & Baseline>PeakAnalyzer function in Origin 2021. A user defined baseline was established using a conservative approach (using ≤ 4 points) and B-spline interpolation. The baseline was then subtracted from the raw data (**Figure S18a**). Finally, a pseudo-Voigt peak was fitted to the (020) peak, as shown in **Figure S18b**.

Real-space values (d) were calculated from q-space values using **Equation S1**:

$$d = \frac{2\pi}{q} \quad \textbf{(S1)}$$

Extent of paracrystalline disorder g [5] was calculated using **Equation S2**:

$$g_{(020)} \approx \frac{1}{2\pi}\sqrt{\Delta q \bullet d_{(020)}} \quad \textbf{(S2)}$$

DFT Calculations. All calculations were run using Gaussian 16 Rev A.03[6] and NBO 6.0[8] Polymers were modeled as 16 repeating thiophene units, and geometries were optimized in the gas phase using ωB97XD/6-31G* where the range separation factor ω was set to 0.1 as a representative value for conjugated polymers. At the minimized geometry, the optimal ω value was found such that Koopmans' theorem was best described using a protocol described in previous work.[9] All subsequent calculations used this optimized ω value. Solvent was modeled using a self-consistent reaction field polarizable continuum.

Additional Discussions

Electrochemistry: Susceptibility to doping was additionally monitored by DPV measured in 0.1 M EMITos/PC. This electrolyte system was chosen so that tosylate (Tos) would serve as the charge-balancing anion in both the solid-state and electrochemical measurements. By DPV (**Figure S3**), P(OE3)-E has the lowest onset of oxidation (-0.80 V vs. Fc/Fc^+) followed by P(OE3)-D (-0.65 V vs. Fc/Fc^+) and P(OE3)-Ph (-0.55 V vs. Fc/Fc^+). The DPV data is in agreement with the UV-vis-NIR data in **Figure 3**, showing that P(OE3)-E oxidizes at lower potentials compared to P(OE3)-D and P(OE3)-Ph. These DPV results are also consistent with oxidation onset trends from DPV and cyclic voltammetry (CV) collected for our previous study, where films were measured in both aqueous and organic electrolytes.[2]

Spectroelectrochemistry: To monitor evolution of the charge carrier optical absorbances, potential-dependent spectra (spectroelectrochemistry) was employed (**Figure S4**). A bathochromic shift of the π-π* transition for P(OE3)-E is consistent with EDOT-incorporation into the copolymer.[10,11] Increasing the potential causes a gradual loss of the π-π* transitions with concomitant appearance of charge carrier bands in the near-IR.[12] Consistent with DPV results, charge carrier bands begin to develop at a lower potential for P(OE3)-E (-0.75 V vs. Fc/Fc^+) relative to P(OE3)-D (-0.55 V vs. Fc/Fc^+) and P(OE3)-Ph (-0.45 V vs. Fc/Fc^+). Additionally, there are notable distinctions in the higher energy charge carrier band peaks developed between 700-1200 nm, with peak maxima observed at different wavelengths and potentials: P(OE3)-E (λ_{max} = 1070 nm at -0.25 V), P(OE3)-D (λ_{max} = 930 nm at -0.15 V), and P(OE3)-Ph (λ_{max} = 900 nm at 0.05 V). Finally, we observe fewer intermediate spectra between the neutral π-π* transition absorption and the fully bleached state of P(OE3)-E relative to the other polymers, corroborating solid-state UV-vis-NIR results (**Figure 3**) which suggest that P(OE3)-E is generally more susceptible to doping.

XPS Analysis: Using XPS, sulfur species can be identified by a characteristic doublet—two peaks that are constrained by spin-orbit splitting and thus are fixed in binding energy (BE), intensity ratio, and full-width at half maximum (FWHM).

First, the pristine spectra for as-cast blade-coated films were deconvoluted to serve as a reference. A single doublet (neutral S-2$p_{3/2}$ and neutral S-2$p_{1/2}$) was initially assigned to the pristine spectra, corresponding to neutral thiophene. This neutral doublet, like all S-2p doublets, is composed of a $p_{3/2}$ and a $p_{1/2}$ peak equal in FWHM, separated by ~1.17 eV, with a

$p_{1/2}$: $p_{3/2}$ intensity ratio of approximately 1:2 (~0.511).[3] These numerical constraints are fixed due to spin-orbit splitting and are consistent with the anticipated chemical species present. However, in the pristine XPS spectra (see **Figures S5a**, **S6a**, **S7a**) a single neutral thiophene doublet results in some residual error at higher binding energies, so a second doublet was assigned (S-2$p_{3/2}$ and S-2$p_{1/2}$).[13,14] To quantify the presence of the sulfur species corresponding to theis second doublet, the area under the doublet of S-2$p_{3/2}$ and S-2$p_{1/2}$ is divided by the total area under the S-2p spectra. Intuititvely, this ratio yields the fraction of the species present in the undoped film relative to the total sulfur abundance. The area ratios of the second doublet to the neutral thiophene doublet of the pristine films are listed in (**Table S3**), with the doublet area ratios being the largest for P(OE3)-E (0.13) followed by P(OE3)-Ph (0.08) and P(OE3)-D (0.06). We note that assigning a specific chemical species to this second doublet (S-2$p_{3/2}$ and S-2$p_{1/2}$) that arises in the pristine polymer is complex as it could be potentially attributed to other polaronic species[15,16] from surface air oxidation or other sulfur species (e.g., sulfone groups)[17] due to photo-degradation[18,19] or excitation processes (e.g., shake up/off, asymmetry).[14,20,21]

Next, the spectra of the doped samples were deconvoluted to obtain carrier ratios, *c*, at each dopant concentration level. S-2p spectra are deconvoluted for the three chemical species present in the chemically doped films: neutral thiohene, oxidized thiophene, and tosylate anions. *c* values were calculated using the following ratios:

$$\frac{A(S_{ox\,thio})}{A(S_{ox\,thio})+A(S_{neut\,thio})}, \frac{A(S_{tosylate})}{A(S_{ox\,thio})+A(S_{neut\,thio})}$$

where $A(S_{ox\,thio})$ is the area of the oxidized thiophene sulfur doublets, $A(S_{neut\,thio})$ is the area of the neutral thiophene sulfur doublets and $A(S_{tosylate})$ is the area of the tosylate sulfur doublets. For the first method, *c* ought to be equal to the area ratio of the oxidized thiophene doublet with respect to the total thiophene doublets (oxidized + neutral).[3] For the second method, *c* ought to be equal to the area ratio of the tosylate (Tos) counterion doublet with respect to the total thiophene doublets, assuming one tosylate counterion per polaronic charge carrier. In using a simple alogrithm, an initial constraint used was that the atomic abundance of $S_{tosylate}$ and $S_{ox\,thio}$ should be equivalent. This constraint is used because there should be a counterion (the tosylate anion) stabilizing every charge formed along the backbkone (oxidized thiophene). The constraint was then removed after subsequent interactions to consider defects or impurity.

To establish confidence in the deconvolution processes, the BE and FWHM values for the doped films are evaluated. Deconvoluting the S-2p thiophene peak into neutral and oxidized contributions, as demonstrated by previous electrochemical and spectroscopic reports, is a valid and a self-consistent means to quantifying the extent of oxidation and doping.[3,22] The average S-2p$_{3/2}$ binding energies differences (ΔBE) between the neutral and oxidized thiophene species for the P(OE3) series at all doping levels is ~0.53 eV (**Table S4**), which is consistent with ΔBE of 0.6-0.9 eV observed for other poly(alkyl thiophenes).[3,22] Additionally, the oxidized thiophene FWHM is consistent across all chemistries and doping levels (**Figure S8**), which indicates that the photoelectron decay process (due to instrumentation and chemical environment)[23] is similar, as should be expected. Consequently, the consistency observed for the oxidized thiophene ΔBE and FWHM values provides a high level of confidence in the deconvolution procedures. Furthermore, tosylate moieties are the counter anions to the polaronic charge carriers; therefore, the characteristics of the tosylate peak in the S-2p spectra must also be considered. The S-2p electrons in the tosylate anion sulfonate group have a higher binding energy relative to neutral and oxidized thiophene, due to presence of three electronegative oxygen atoms adjacent to the sulfur atom.[24,25] Like the oxidized thiophene moieties, the tosylate deconvolutions have similar ΔBE and FWHM values (**Figure S8**), independent of the doping level and P(OE3) polymer chemistry, and this consistency provides a high level of confidence in the deconvolution procedures.

The SLoT Model and Charge Transport Analysis of W_H: W_{H} is the localization energy, which can be represented by the depth of a potential energy well that localizes carriers, and this localization arises from spatial and electrostatic barriers (e.g., Columbic interactions with counteranions, microstructural inhomogeneity, bond order perturbation and polarization). Notably, W_{H} decreases as c increases as these potential wells impinge and overlap with one another. In some material systems at high carrier ratios, $W_{\mathrm{H}}(c)$ can become $\leq k_{\mathrm{B}}T$; in this regime, the effects of localization are minimal, and transport can be considered delocalized.[14] As W_H and c are experimentally measurable, the rate at which localization decreases with increasing carrier ratio can be calculated.[26]

In the main text, we evaluate the σ_0 and $\eta(c)$ expressions because they best explain why these P(OE3) copolymer obtain different transport properties. Here, we turn to examining the $W_{\mathrm{H}}(c)$ relationship, the localization energy as a function of carrier ratio for a holistic SLoT analysis. To evaluate this relationship, temperature-dependent thermoelectric measurements and carrier concentration ratio measurements are needed. **Figure S20** shows that W_{H} is shown

to decrease with increasing c in all systems. The $W_{\mathrm{H}}(c)$ curves have different slopes and intercepts with the minimal localization energy (denoted by the horizontal dashed line), suggesting that the P(OE3) comonomer selection can affect the spatial and electrostatic localization of charge carriers. Although the $W_{\mathrm{H}}(c)$ curves are significantly different, the W_{H} values and curves converge in the high-doping regime ($c > 0.3$) to 20-40 meV; therefore, W_{H} cannot sufficiently explain the differences in conductivity in this comparison study.

Additional Figures

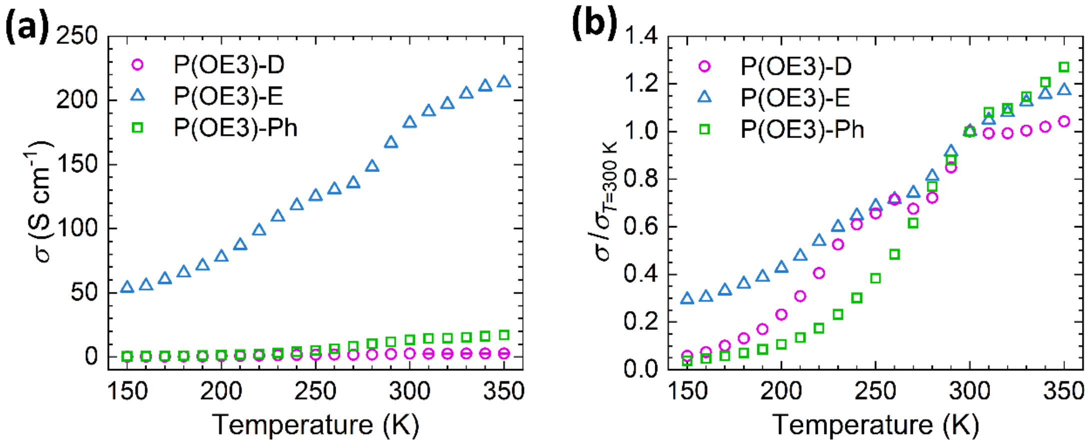

Figure S1. (a) Representative temperature-dependent electrical conductivity (σ) measurements of blade-coated P(OE3) films doped with 5 mM $FeTos_3$. (b) Normalized temperature-dependent σ data of the P(OE3) series (normalized to the $\sigma_{T=300K}$).

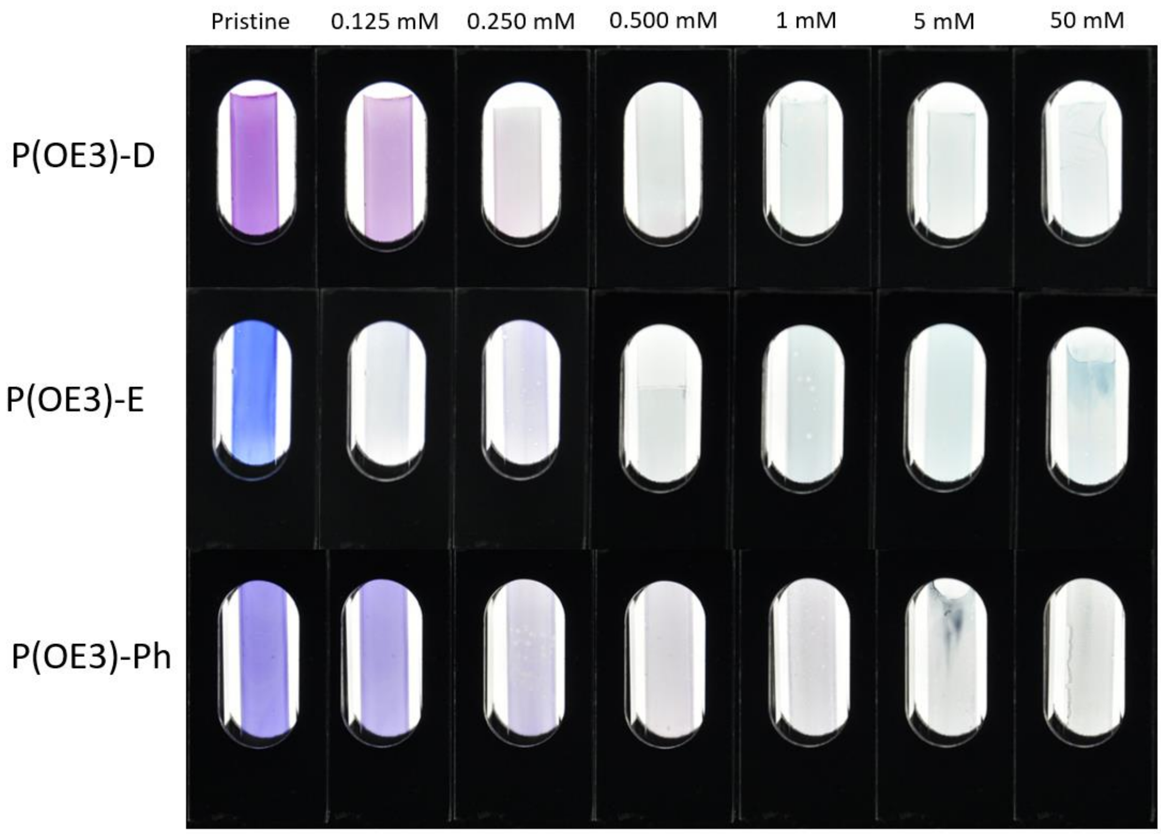

Figure S2. Photos of blade-coated films doped to different concentrations (pristine, 0.125 mM, 0.250 mM, 0.500 mM, 1 mM, 5 mM, 50 mM) of $FeTos_3$/ACN at a doping time of 1 minute. Note: as the as-cast blade-coated P(OE3)-E films demonstrated significant oxidation in air (consistent with our previous study[2]), P(OE3)-E films were treated with hydrazine vapors to achieve a similarly reduced state to the as-cast blade-coated P(OE3)-D and P(OE3)-Ph films, prior to being chemically doped with FeTos3.

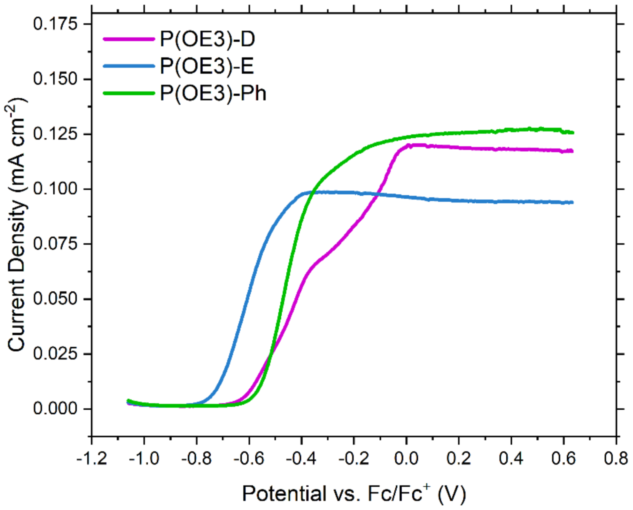

Figure S3. Differential pulse voltammograms of P(OE3) polymers in 0.1 M EMITos/PC (step size 2 mV, step time 0.1 s, pulse amplitude 10 mV, and pulse time 0.02 s).

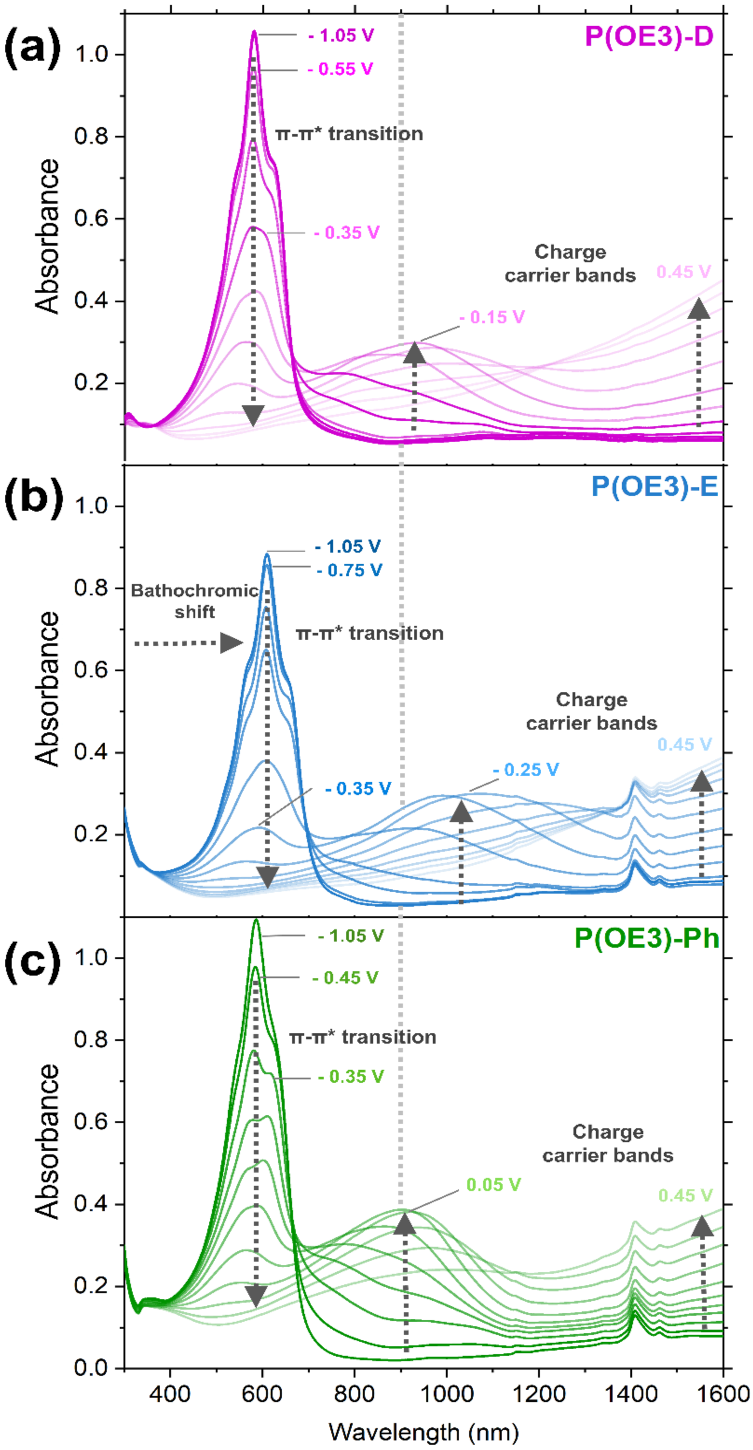

Figure S4. Spectroelectrochemistry of (a) P(OE3)-D, (b) P(OE3)-E, (c) P(OE3)-Ph films on ITO/glass recorded every 100 mV in 0.1 M EMI-Tos/PC from -1.05 V to +0.45 V vs. Fc/Fc^{+}. To aid visualization of higher energy carrier band peaks, a dotted grey line has been dropped at 900 nm.

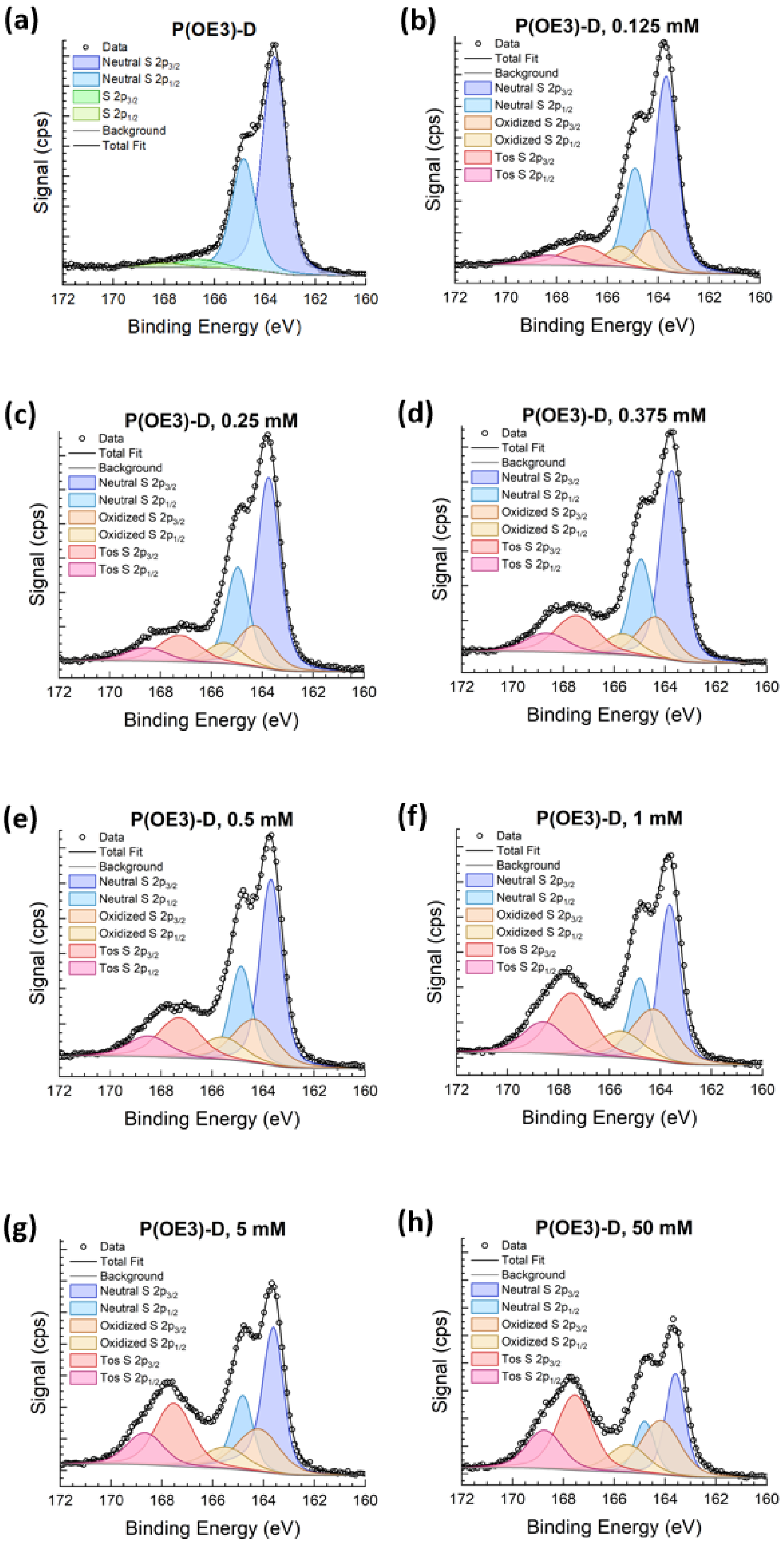

Figure S5. XPS S-2p spectra for P(OE3)-D: (a) pristine, (b) 0.125 mM, (c) 0.25 mM, (d) 0.375 mM, (e) 0.5 mM, (f) 1 mM, (g) 5 mM, (h) 50 mM $FeTos_3$/ACN.

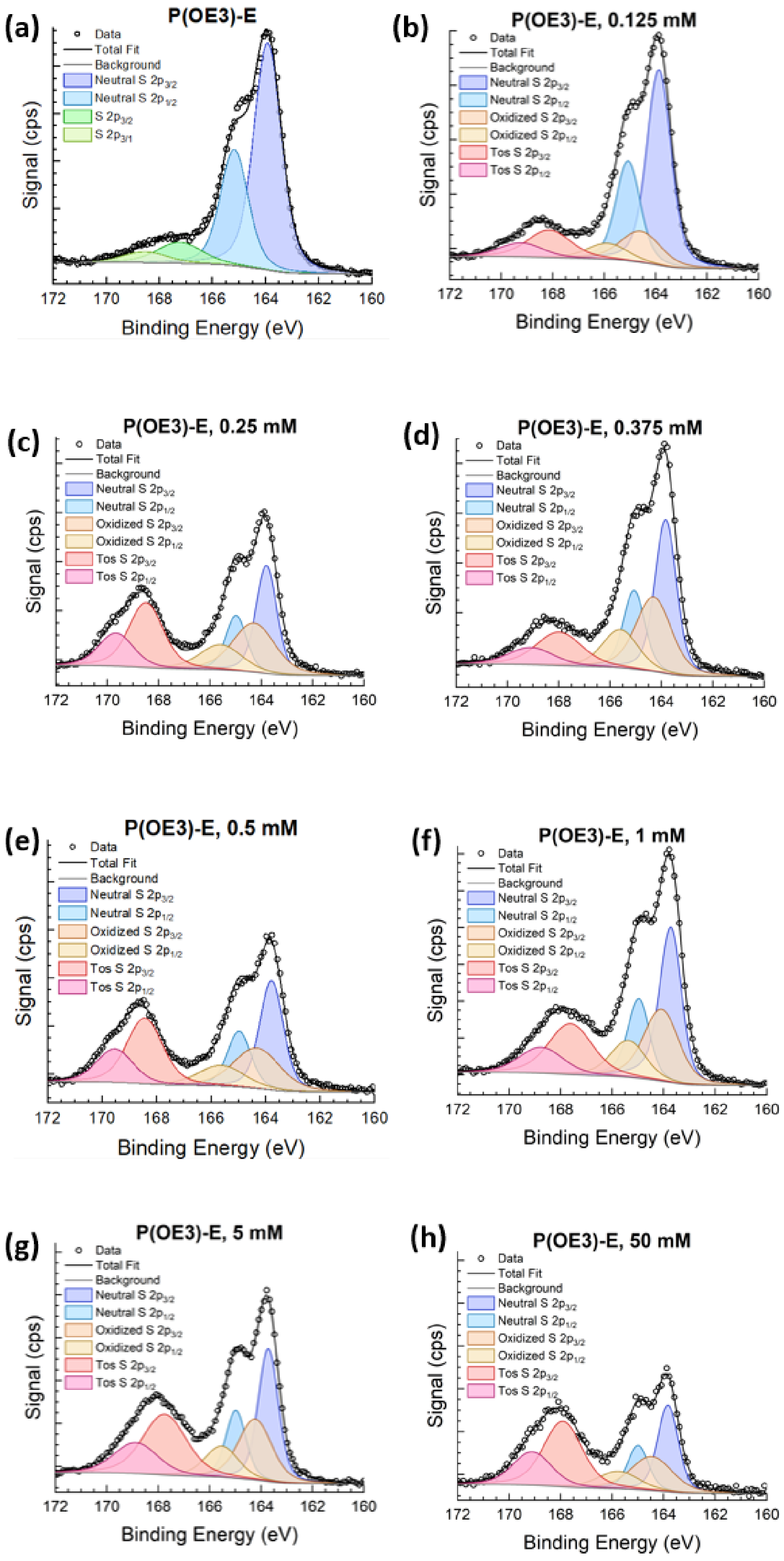

Figure S6. XPS S-2p spectra for P(OE3)-E: (a) pristine, (b) 0.125 mM, (c) 0.25 mM, (d) 0.375 mM, (e) 0.5 mM, (f) 1 mM, (g) 5 mM, (h) 50 mM $FeTos_3$/ACN.

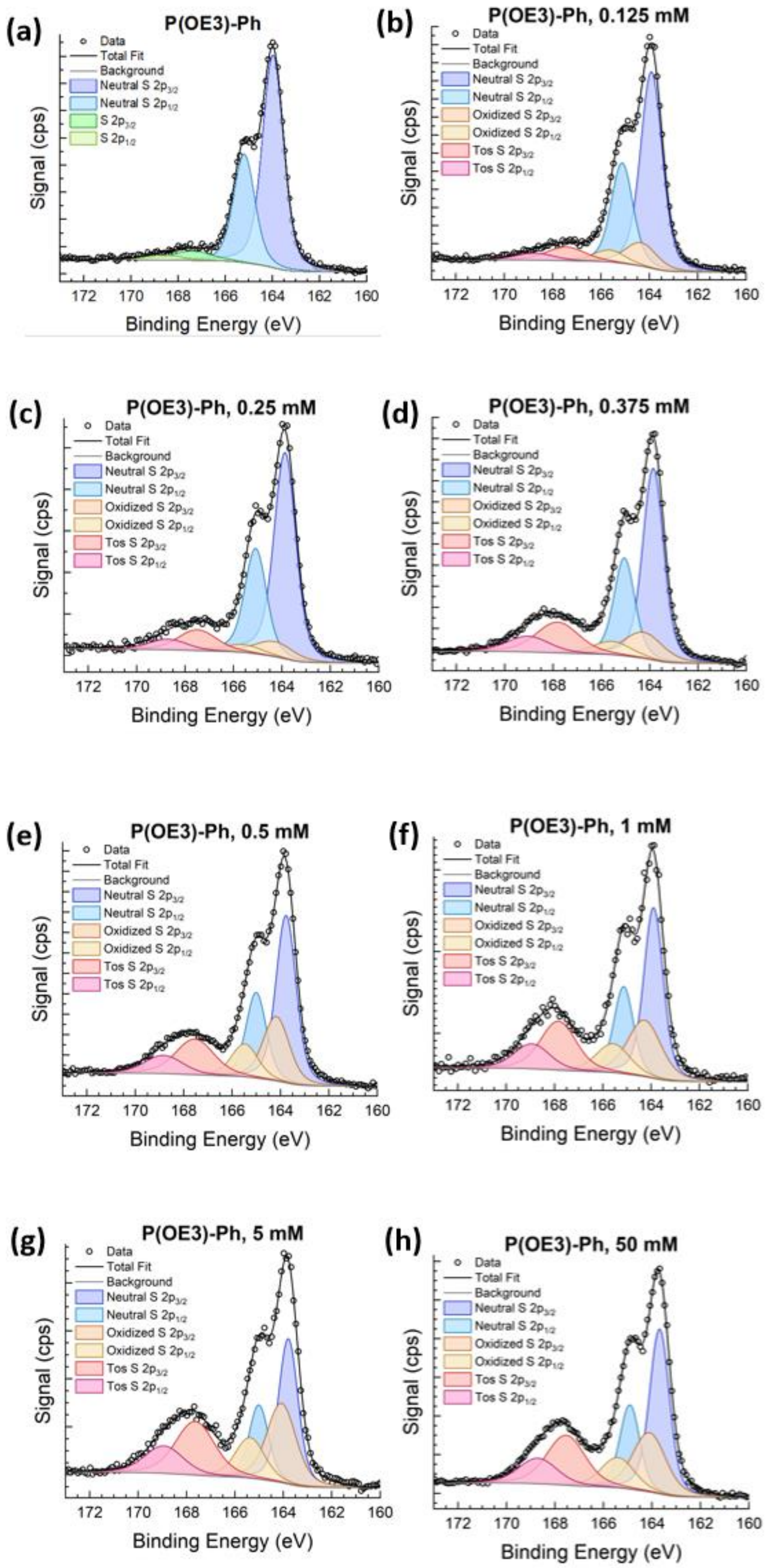

Figure S7. XPS S-2p spectra for P(OE3)-Ph: (a) pristine, (b) 0.125 mM, (c) 0.25 mM, (d) 0.375 mM, (e) 0.5 mM, (f) 1 mM, (g) 5 mM, (h) 50 mM $FeTos_3$/ACN.

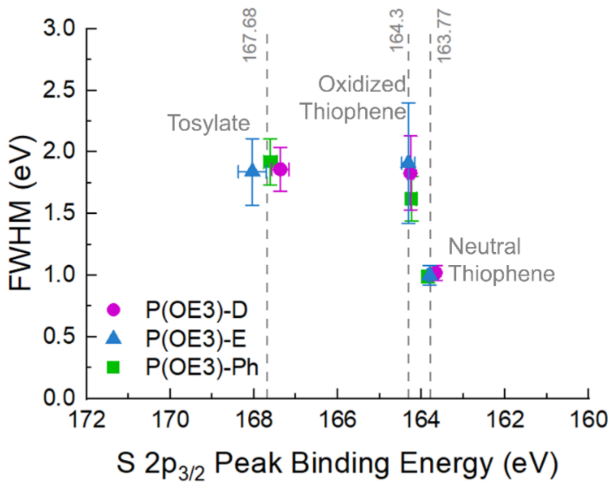

Figure S8. XPS average and standard deviations of S-2p$_{3/2}$ peak fits for each polymer.

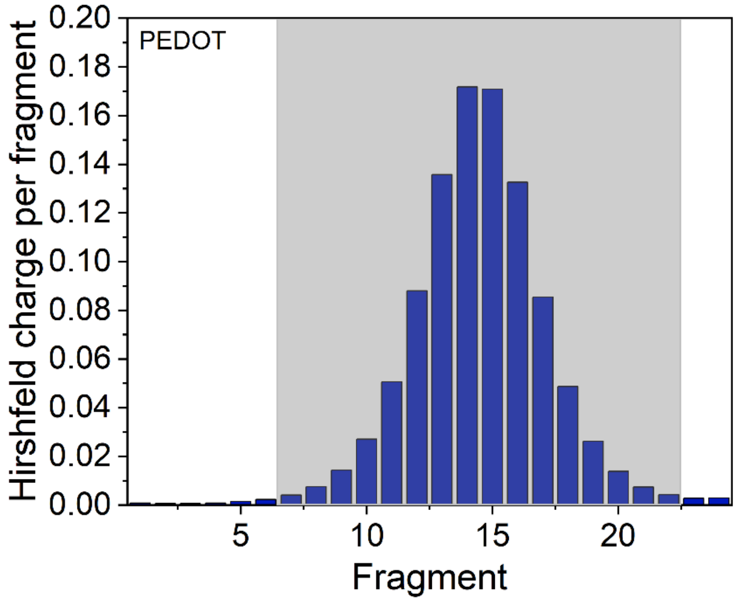

Figure S9. Hirshfeld charge per fragment approximating charge delocalization over a test 24-mer PEDOT in an oxidized state. Values were calculated by DFT (ωB97XD/6-31G*).

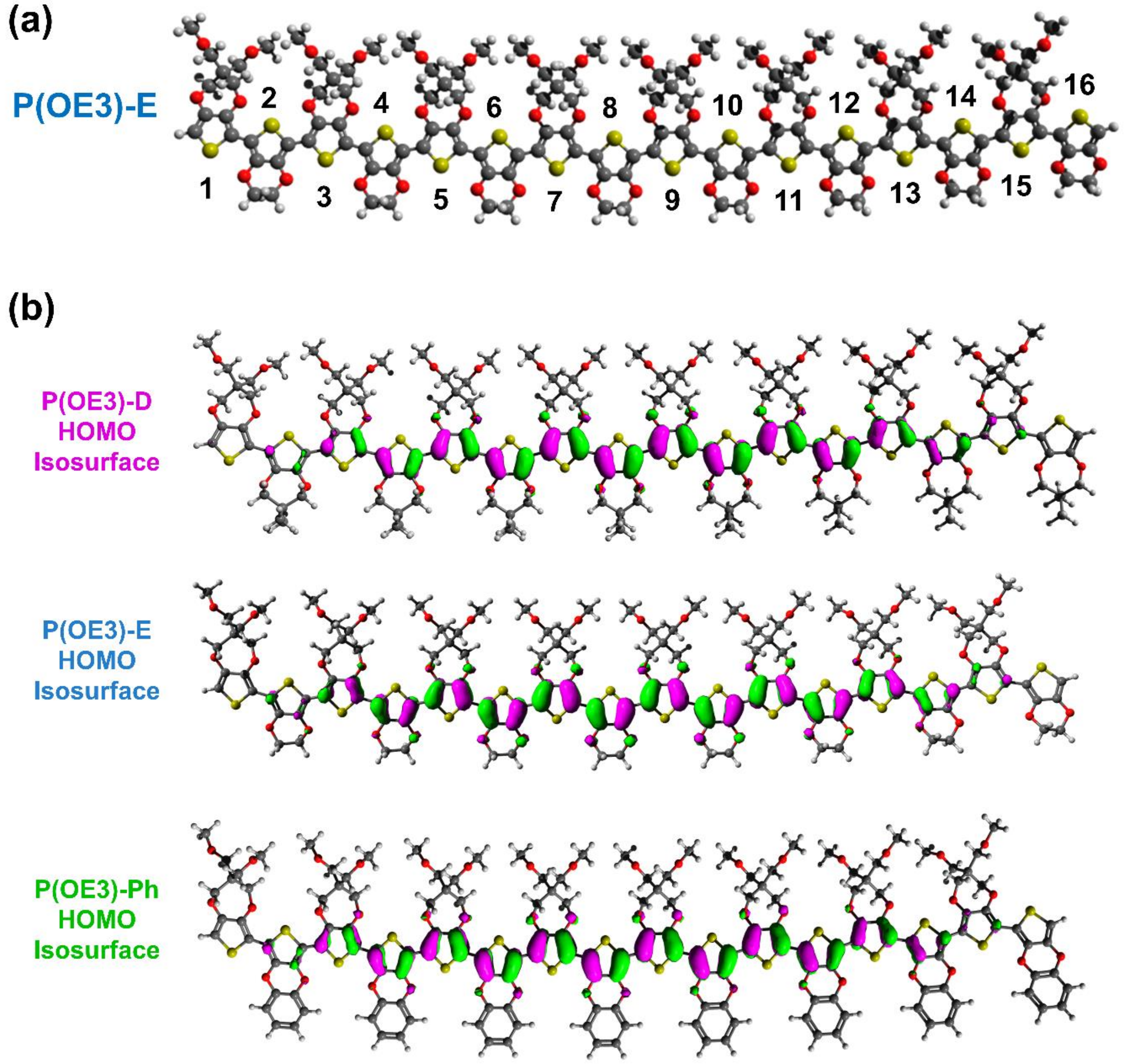

Figure S10. DFT calculations (ωB97XD/6-31G*) for the P(OE3) series. (a) Representative example of a geometry optimized hexadecamer structure [in this case P(OE3)-E]. Rings ("fragments") are labeled 1 through 16 for clarity. (b) Isosurfaces of the highest occupied molecular orbitals (HOMOs) for each polymer.

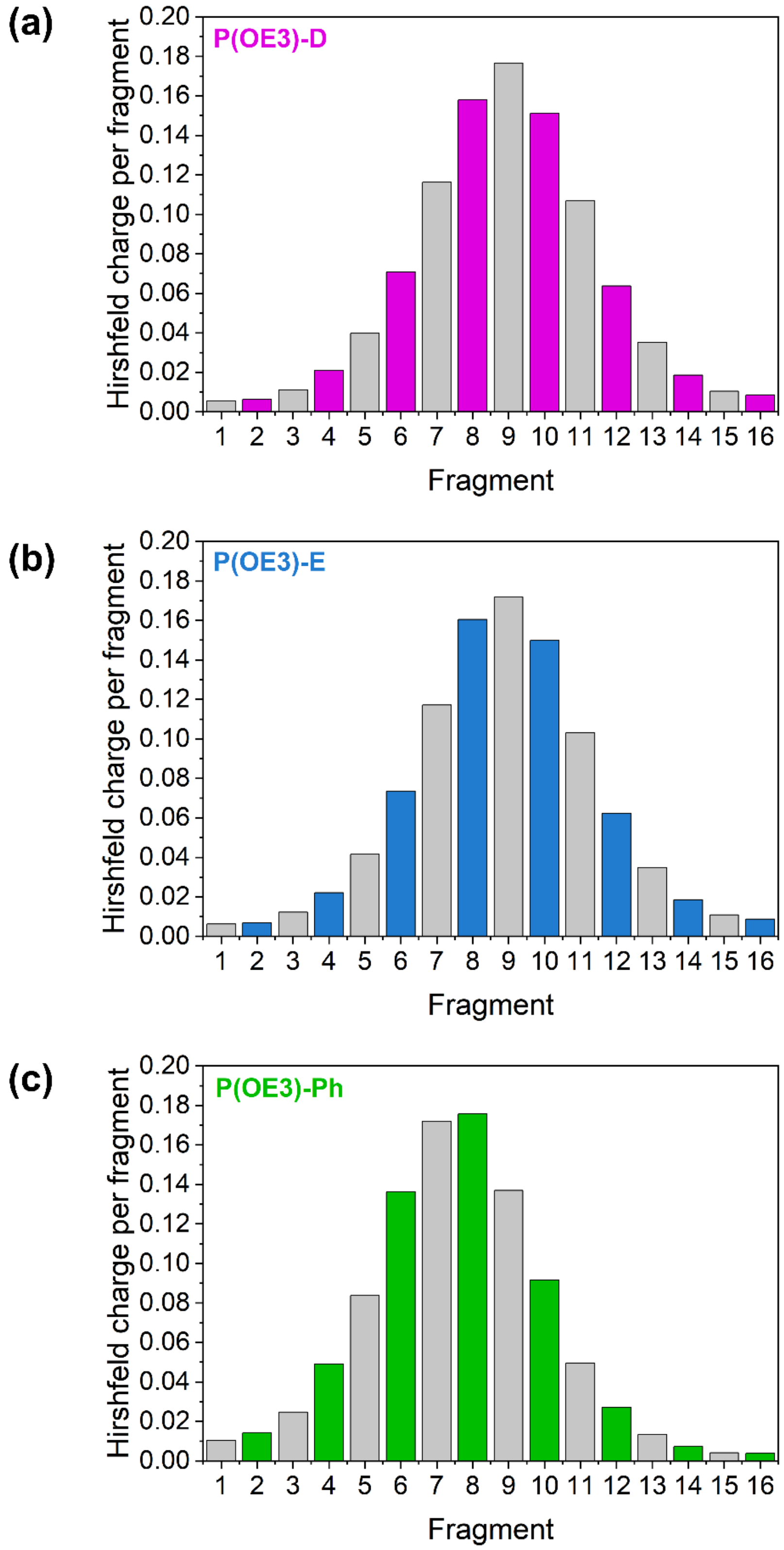

Figure S11. Calculated Hirshfeld charge per fragment of the hexadecamer to approximate the charge delocalization of the polymer in the oxidized state. Fragments 1,3,5,7,9,11,13,15 (shaded in gray) are the oligoether-functionalized ProDOT units, P(OE3). Fragments 2,4,6,8,10,12,14,16 (shaded with vibrant colors) are the comonomer units.

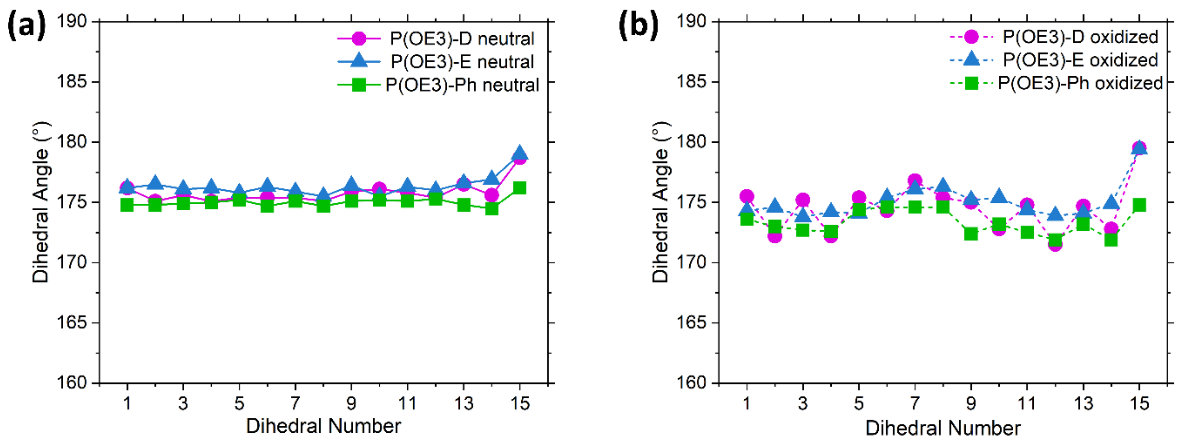

Figure S12. Dihedral angles (°) calculated using model dodecamer structures in the (a) neutral and (b) oxidized state. Dihedral number indicates relative position along the dodecamer.

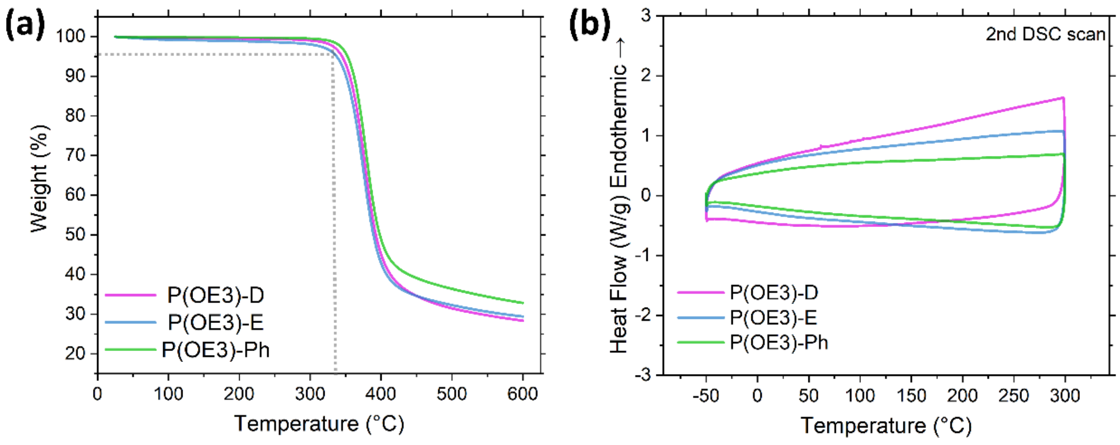

Figure S13. Thermal characterization of the P(OE3) series. All measurements run under an inert argon atmosphere. (a) TGA taken from 30 °C to 600 °C at a scan rate of 10 °C/minute. Horizontal and vertical dotted lines mark the 95 weight % and corresponding temperature, respectively. (b) DSC scans taken between -50 °C to 300 °C at a scan rate of 10 °C/minute. 2nd DSC scan shown.

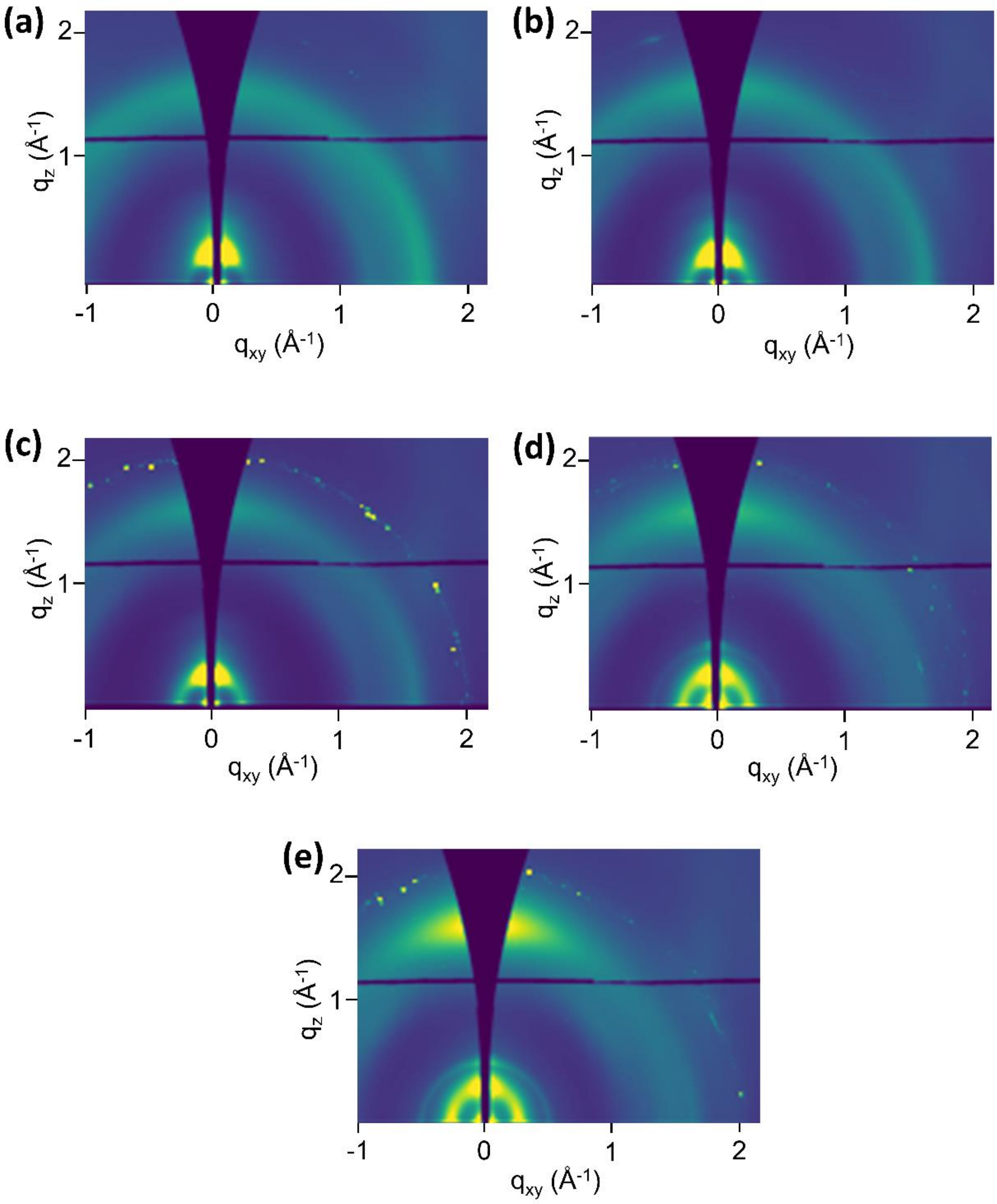

Figure S14. GIWAXS diffractograms of (a) pristine P(OE3)-D and P(OE3)-D doped with (b) 0.125 mM, (c) 0.5 mM, (d) 5 mM, and (e) 50 mM $FeTos_3$/ACN.

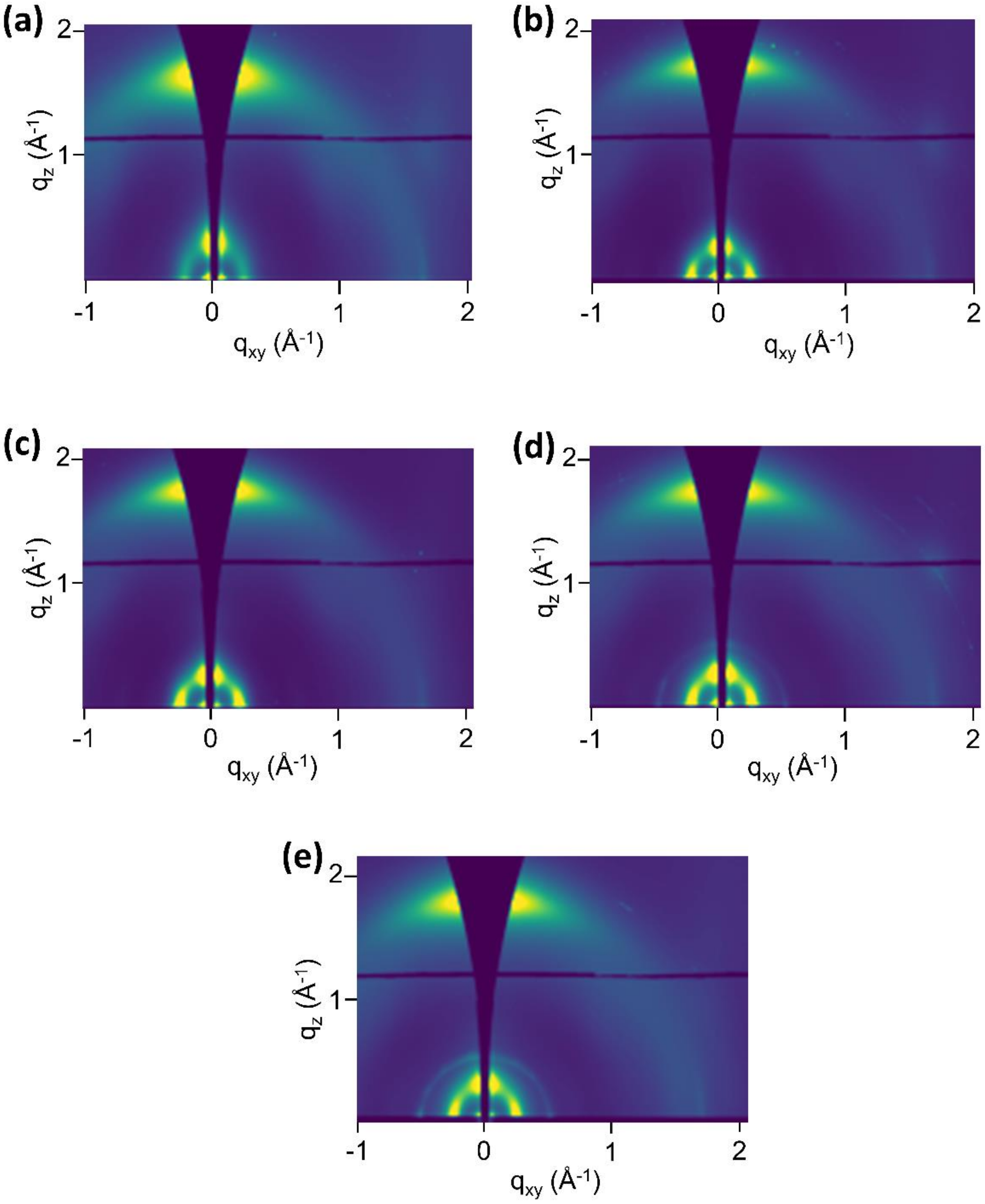

Figure S15. GIWAXS diffractograms of (a) pristine P(OE3)-E and P(OE3)-E doped with (b) 0.125 mM, (c) 0.5 mM, (d) 5 mM, and (e) 50 mM $FeTos_3$/ACN.

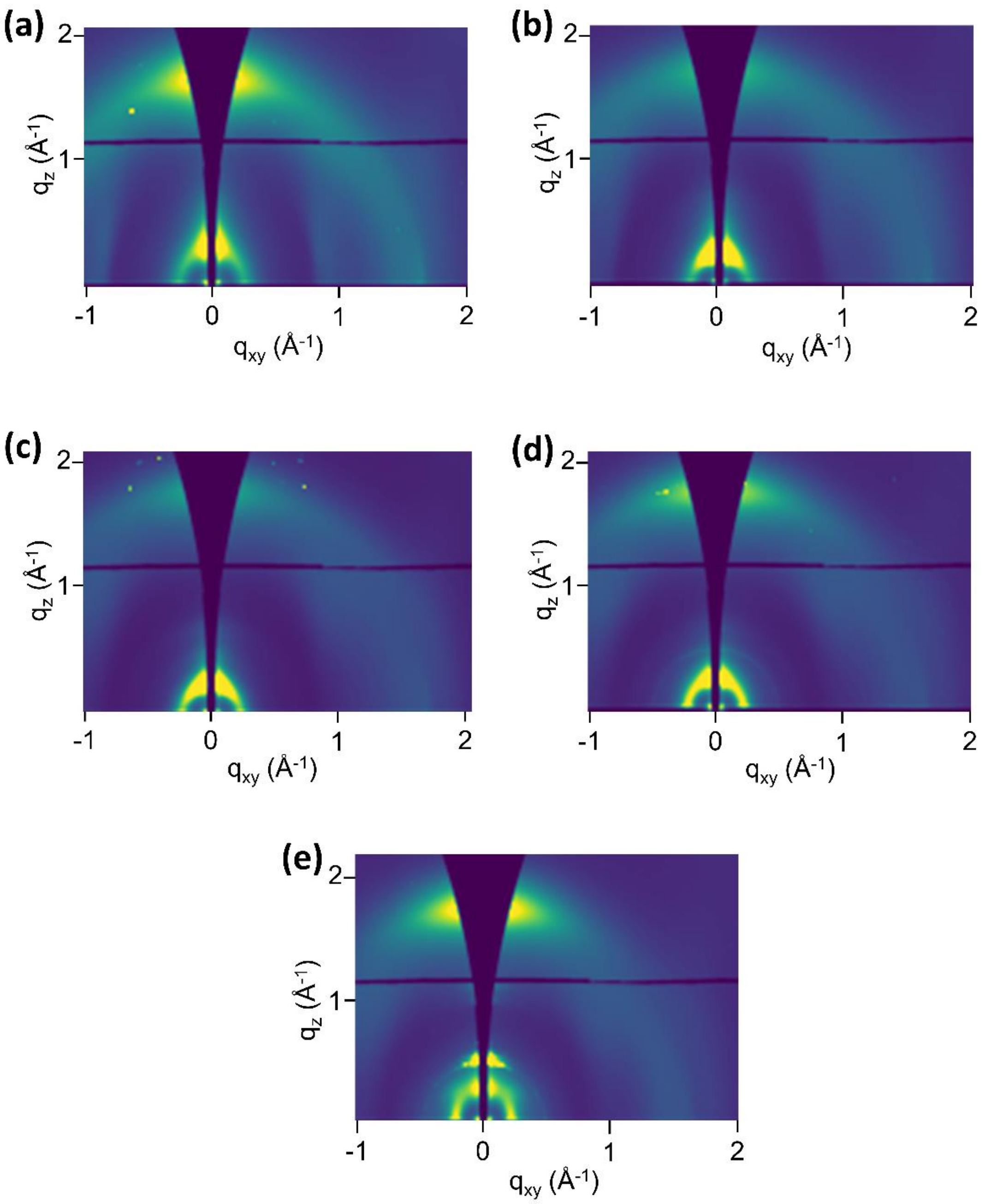

Figure S16. GIWAXS diffractograms of (a) pristine P(OE3)-Ph and P(OE3)-Ph doped with (b) 0.125 mM, (c) 0.5 mM, (d) 5 mM, and (e) 50 mM $FeTos_3$/ACN.

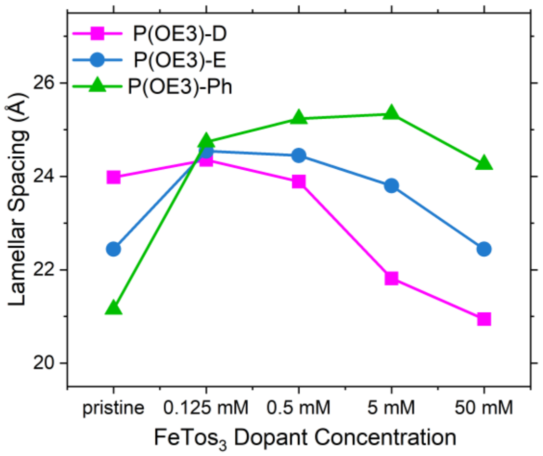

Figure S17. Lamellar spacings calculated from the GIWAXS profiles as a function of dopant concentration.

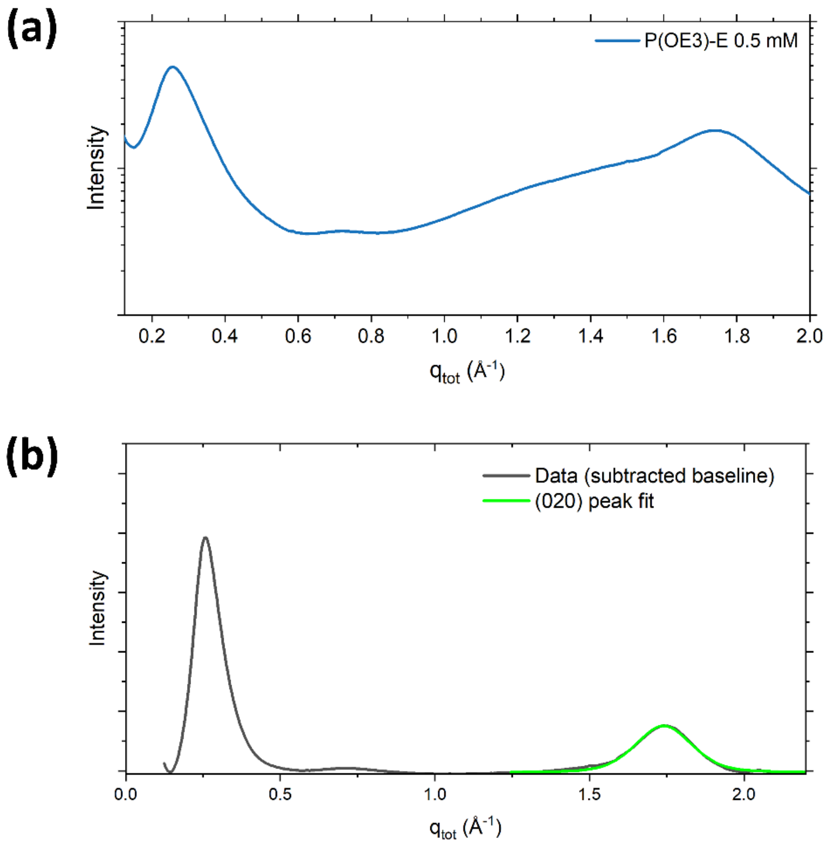

Figure S18. (a) Radially integrated GIWAXS profile of 0.5 mM $FeTos_3$ doped film of P(OE3)-E. (b) Deconvoluted scattering peaks from the radially integrated GIWAXS profile.

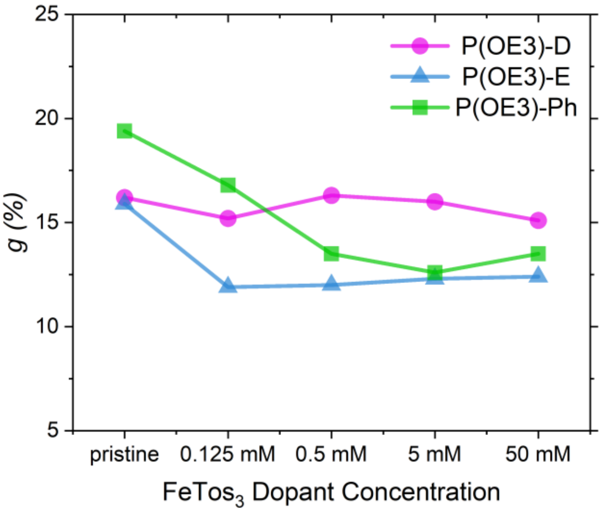

Figure S19. *g* parameter values calculated from the GIWAXS profiles as a function of dopant concentration.

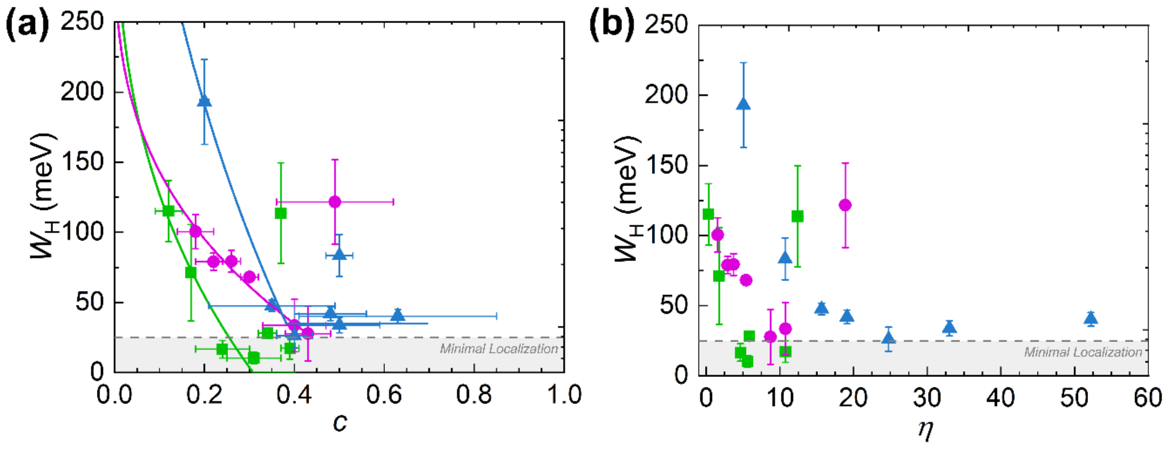

Figure S20. Analysis of the localization energy. (a) Localization energies as a function of the carrier concentration ratio. Solid lines represent $W_H(c)$ model curves. Activation energy is minimal when $W_H < k_BT$, and transport is thermally deactivated when $W_H < 0$. Note that W_H generally decreases as c increases, except data points near the coordinate (0.5, 100 meV). In the case of P(OE3)-D and P(OE3)-Ph, these data points are at 50 mM $FeTos_3$ and may indicate over-doping. In the case of P(OE3)-E, this data point is a result of the high c value calculated from XPS; the trend is more obvious in the $W_H(\eta)$ plot. (b) Localization energy as a function of reduced Fermi energy level.

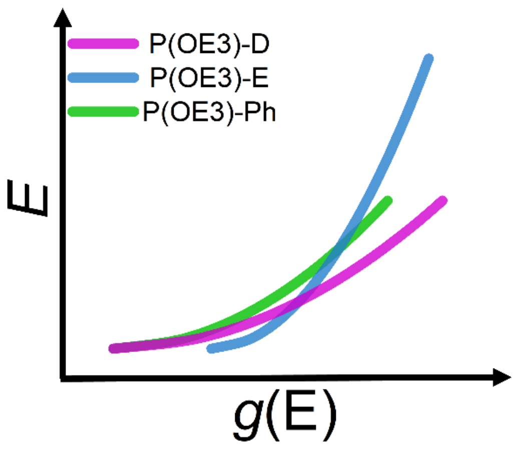

Figure S21. Cartoon schematically illustrates a possible density of electronic states for the P(OE3) series as suggested by differences in c_t values and $\eta(c)$ values.

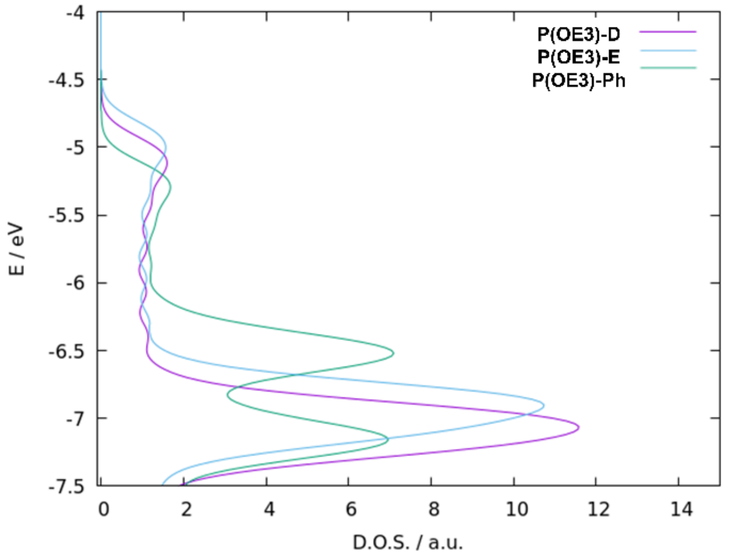

Figure S22. The density of states (D.O.S.) calculated for the hexadecamers by DFT calculations (ωB97XD/6-31G*).

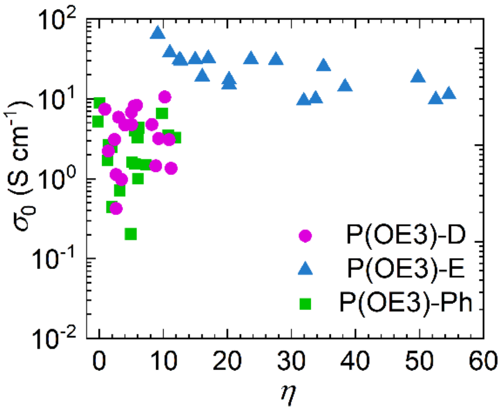

Figure S23. σ_0 as a function of η. Eq. 1-3 show that σ_0 is the transport function constant and can be calculated by dividing σ_{E_0} by the localization energy, calculated from temperature-dependent thermoelectric measurements.[26] Within a polymer system, we calculate that σ_0 does not have a statically significant dependence on η and that P(OE3)-E has a significantly larger σ_0 than P(OE3)-D and P(OE3)-Ph.

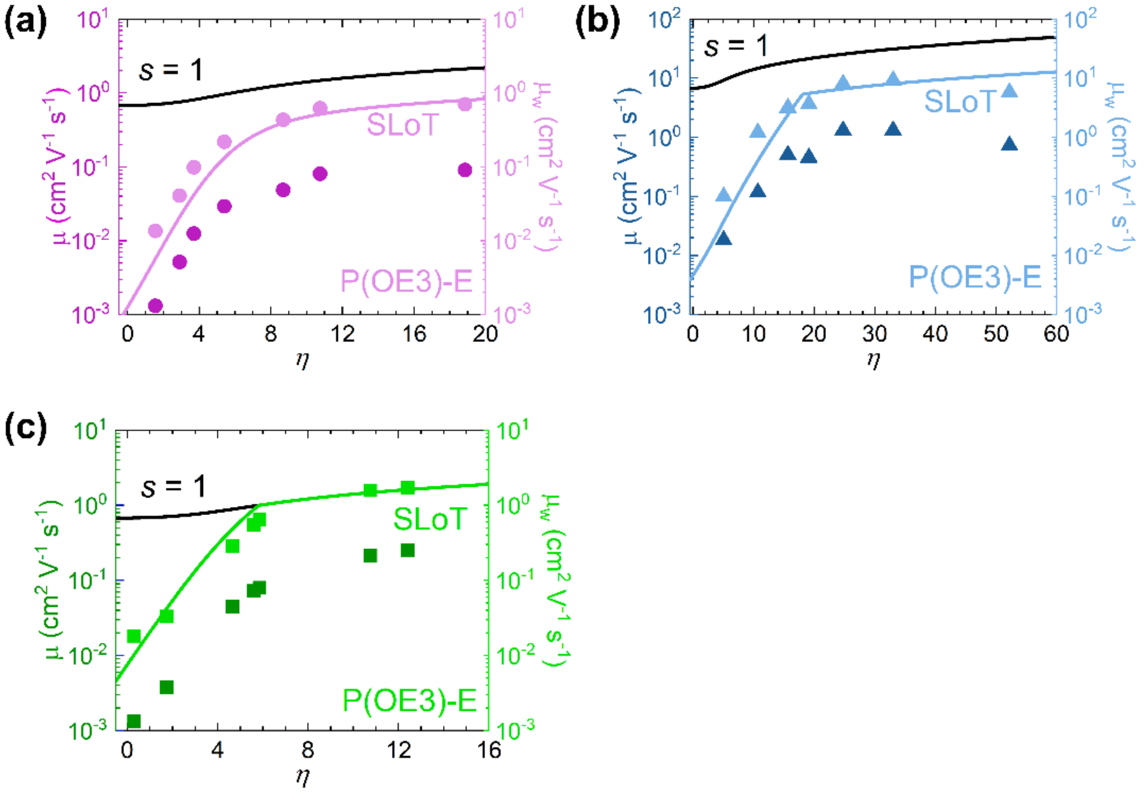

Figure S24. Analyzing carrier mobilities. (a) P(OE3)-D. (b) P(OE3)-E. (c) P(OE3)-Ph. In all plots, the darker colored points represent the doping-level average drift (Drude) mobility, and the lighter colored data points represent the doping-level average weighted mobility.[27] Both mobility calculations indicate that the mobility increases with increasing doping level and then begins to plateau. Additionally, all plots show the weighted mobility model fits calculated using the Kang-Snyder $s = 1$ model (with a constant σ_{E_0} value) and the SLoT model (where σ_{E_0} increases as localization decreases).[26,28] Note that the SLoT model provides a better fit. Lastly, note that the scalar difference between μ and μ_w is indicative of the weighted mobility effective mass, as shown in Table S7.

Additional Tables

Table S1. Measured thicknesses of blade-coated films doped with $FeTos_3$.

Concentration $FeTos_3$ (mM)	P(OE3)-D (nm)	P(OE3)-E (nm)	P(OE3)-Ph (nm)
0.125	179, 227, 171	958, 861, 1164	352, 675, 379
0.25	202, 199, 250	933, 1080, 861	534, 268, 404
0.375	200, 198, 214	729, 719, 766	745, 460, 463
0.5	200, 222, 204	755, 764, 726	206, 375, 347
1	224, 225, 235	389, 662, 898	638, 578, 530
5	267, 264, 235	443, 915, 1300	323, 661, 653
50	262, 281, 221	960, 1270, 1560	655, 266, 652

Table S2. Measured electrical conductivities of the P(OE3)-series.

Concentration $FeTos_3$ (mM)	P(OE3)-D ($S\ cm^{-1}$)	P(OE3)-E ($S\ cm^{-1}$)	P(OE3)-Ph ($S\ cm^{-1}$)
0.125	0.066 ± 0.059	1.09 ± 0.84	0.045 ± 0.018
0.25	0.317 ± 0.140	17.5 ± 5.6	0.177 ± 0.084
0.375	0.905 ± 0.341	52.7 ± 3.9	3.00 ± 1.69
0.5	2.45 ± 0.34	64.7 ± 12.5	5.41 ± 0.81
1	5.86 ± 0.86	156 ± 43	7.51 ± 1.31
5	9.02 ± 0.95	195 ± 26	23.0 ± 1.8
50	12.4 ± 0.9	137 ± 26	26.1 ± 2.1

Table S3. Ratio of second doublet population (*ca.* 167.1 eV).

**Polymer**	Ratio of second doublet area (S-$2p_{3/2}$ and S-$2p_{1/2}$) to primary doublet (neutral S-$2p_{3/2}$ and neutral S-$2p_{1/2}$)
P(OE3)-D	0.06
P(OE3)-E	0.13
P(OE3)-Ph	0.08

Table S4. Calculated ΔBE in shifts.

**Polymer**	ΔBE from neutral thiophene to oxidized thiophene (eV)
P(OE3)-D	0.64 ± 0.15
P(OE3)-E	0.52 ± 0.12
P(OE3)-Ph	0.41 ± 0.08

Table S5. Charge ratios and approximate carriers per thiophene ring.

Concentration $FeTos_3$ (mM)	P(OE3)-D		P(OE3)-E		P(OE3)-Ph	
	c	Carriers per thiophene	*c*	Carriers per thiophene	*c*	Carriers per thiophene
0.125	0.18 ± 0.04	1 per 5	0.20 ± 0.01	1 per 5	0.12 ± 0.03	1 per 10
0.25	0.22 ± 0.04	1 per 4	0.50 ± 0.03	1 per 2	0.17 ±0.01	1 per 5
0.375	0.26 ± 0.02	1 per 4	0.35 ± 0.14	2 per 5	0.24 ± 0.06	1 per 4
0.5	0.30 ± 0.02	1 per 3	0.48 ± 0.08	1 per 2	0.31 ± 0.06	1 per 3
1	0.43 ± 0.05	2 per 5	0.40 ± 0.04	2 per 5	0.34 ± 0.02	2 per 5
5	0.40 ± 0.07	2 per 5	0.50 ± 0.09	1 per 2	0.39 ± 0.02	2 per 5
50	0.49 ± 0.13	1 per 2	0.63 ± 0.22	3 per 5	0.37 ± 0.01	2 per 5

Table S6. Onsets of oxidation and ionization energies (IE) of the P(OE3) series.

**Polymer**	E_{ox} vs. Fc/Fc^+ (V)	IE_{theor} (eV)^b
P(OE3)-D	-0.65	4.41
P(OE3)-E	-0.80	4.29
P(OE3)-Ph	-0.55	4.59

^{a}Onsets of oxidation estimated from the DPV data in Figure S3.
^{b}IE calculated as the difference in electronic energy of a radical cation and neutral state of a representative hexadecamer structure.

Table S7. Summation of unit contributions to the Hirshfeld polaron charge distributions.

**Polymer**	P(OE3)	Comonomer
P(OE3)-D	50%	50%
P(OE3)-E	50%	50%
P(OE3)-Ph	49%	51%

Table S8. q-values, d-values, and paracrystallinity values extracted from radially integrated line cuts.

**P(OE3)-D**	(100) lamellar spacing		(020) π-π spacing			
	q (Å^{-1})	*d* (Å)	*q* (Å^{-1})	*d* (Å)	Δ_q (Å^{-1})	*g* (%)
Pristine	0.262	24.0	1.53	4.11	0.252	16.2
0.125 mM	0.258	24.4	1.56	4.03	0.225	15.2
0.5 mM	0.263	23.9	1.58	3.98	0.263	16.3
5 mM	0.288	21.8	1.58	3.98	0.254	16.0
50 mM	0.300	20.9	1.59	3.95	0.228	15.1
**P(OE3)-E**	(100) lamellar spacing		(020) π-π spacing			
	q (Å^{-1})	*d* (Å)	*q* (Å^{-1})	*d* (Å)	Δ_q (Å^{-1})	*g* (%)
Pristine	0.280	22.4	1.66	3.79	0.264	15.9
0.125 mM	0.256	24.5	1.74	3.61	0.156	11.9
0.5 mM	0.257	24.4	1.74	3.61	0.157	12.0
5 mM	0.264	23.8	1.73	3.63	0.165	12.3
50 mM	0.280	22.4	1.75	3.59	0.170	12.4
**P(OE3)-Ph**	(100) lamellar spacing		(020) π-π spacing			
	q (Å^{-1})	*d* (Å)	*q* (Å^{-1})	*d* (Å)	Δ_q (Å^{-1})	*g* (%)
Pristine	0.297	21.2	1.63	3.85	0.385	19.4
0.125 mM	0.254	24.7	1.69	3.72	0.299	16.8
0.5 mM	0.249	25.2	1.75	3.59	0.201	13.5
5 mM	0.248	25.3	1.76	3.57	0.176	12.6
50 mM	0.259	24.3	1.76	3.57	0.203	13.5

Table S9: SLoT Transport parameter comparisons. All systems doped with $FeTos_3$.

System	c_d [a] -	n_d [a] cm^{-3} x 10^{20}	c_t [b] -	n_t [b] cm^{-3} x 10^{20}	σ_0 [c] $S\ cm^{-1}$	μ_w [d] $cm^2\ V^{-1}\ s^{-1}$	μ_d [e] $cm^2\ V^{-1}\ s^{-1}$	m_w^* [f] -
**P(OE3)-D**	0.56	9.8	0.072	1.3	2	0.70	0.091	4.1
**P(OE3)-E**	-	-	0.16	2.9	20	9.2	1.3	3.7
**P(OE3)-Ph**	0.28	4.9	0.11	1.9	2	1.7	0.25	4.1

[a] c_d is the carrier ratio and n_d is the density needed for $W_H \sim 0$.

Note that these carrier ratios and densities for P(OE3)-D are extrapolated and likely not physically obtainable with this system. Note that these carrier ratios and densities are challenging to assert for P(OE3)-E as its localization plateaus with increasing doping level and plateaus at comparatively large values greater than $k_B T$ (see Figure S15).

[b] c_t is the carrier ratio and n_t is the carrier density needed for the reduced Fermi energy level to be equal to the transport edge.

[c] σ_0 is the energy- and temperature-independent transport function prefactor. This tabulated value is most consistent with both the standard deviation for σ_0 calculated from each set of polymer films and the σ_0 value needed to fit the $S(\sigma)$ and $\mu(\eta)$ curves.

[d] μ_w is the largest weighted mobility, averaged at a doping level.

[e] μ_d is the largest drift mobility, averaged at a doping level.

[f] m_w^* is the weighted mobility effective mass, averaged over all doping levels.

References

(1) Jacobs, I. E.; Moulé, A. J. Controlling Molecular Doping in Organic Semiconductors. *Adv. Mater.* **2017**, *29*, 1703063. https://doi.org/10.1002/adma.201703063.

(2) Advincula, A. A.; Jones, A. L.; Thorley, K. J.; Anna, M. O.; Ponder, J. F.; Reynolds, J. R.; Österholm, A. M.; Ponder, J. F. Probing Comonomer Selection Effects on Dioxythiophene-Based Aqueous-Compatible Polymers for Redox Applications. *Chem. Mater.* **2022**, *34*, 4633–4645. https://doi.org/10.1021/acs.chemmater.2c00511.

(3) Shallcross, R. C.; Stubhan, T.; Ratcliff, E. L.; Kahn, A.; Brabec, C. J.; Armstrong, N. R. Quantifying the Extent of Contact Doping at the Interface between High Work Function Electrical Contacts and Poly(3-Hexylthiophene) (P3HT). *J. Phys. Chem. Lett.* **2015**, *6*, 1303–1309. https://doi.org/10.1021/acs.jpclett.5b00444.

(4) Pandolfi, R. J.; Allan, D. B.; Arenholz, E.; Barroso-luque, L.; Campbell, S. I.; Caswell, T. A.; Blair, A.; Carlo, F. de; Fackler, S.; Fournier, A. P.; Freychet, G.; Fukuto, M.; Kline, R. J.; Li, R.; Liman, C.; Marchesini, S. Computer Programs Xi-Cam: A Versatile Interface for Data Visualization and Analysis. *J. Synchrotron Radiat.* **2018**, *25*, 1261–1270. https://doi.org/10.1107/S1600577518005787.

(5) Rivnay, J.; Noriega, R.; Kline, R. J.; Salleo, A.; Toney, M. F. Quantitative Analysis of Lattice Disorder and Crystallite Size in Organic Semiconductor Thin Films. *Phys. Rev. B Condens. Matter* **2011**, *84*, 045203. https://doi.org/10.1103/PhysRevB.84.045203.

(6) Frisch, M. J. et al. Gaussian 16, Revision A.03. Gaussian, Inc.: Wallingford, CT 2016.

(7) Bryan, A. M.; Santino, L. M.; Lu, Y.; Acharya, S.; D’Arcy, J. M. Conducting Polymers for Pseudocapacitive Energy Storage. *Chem. Mater.* **2016**, *28*, 5989–5998. https://doi.org/10.1021/acs.chemmater.6b01762.

(8) Glendening, E. D.; Landis, C. R.; Weinhold, F. NBO 6.0: Natural Bond Orbital Analysis Program. *J. Comput. Chem.* **2013**, *34*, 1429–1437. https://doi.org/10.1002/jcc.23266.

(9) Moser, M.; Thorley, K. J.; Moruzzi, F.; Ponder, J. F.; Maria, I. P.; Giovannitti, A.; Inal, S.; McCulloch, I. Highly Selective Chromoionophores for Ratiometric Na+ Sensing Based on an Oligoethyleneglycol Bridged Bithiophene Detection Unit. *J. Mater. Chem. C* **2019**, *7*, 5359–5365. https://doi.org/10.1039/c8tc06000b.

(10) Ponder, J. F.; Österholm, A. M.; Reynolds, J. R. Designing a Soluble PEDOT Analogue without Surfactants or Dispersants. *Macromolecules* **2016**, *49* (6), 2106–2111. https://doi.org/10.1021/acs.macromol.5b02638.

(11) Liu, Z.; Hu, Y.; Li, P.; Wen, J.; He, J.; Gao, X. Enhancement of the Thermoelectric Performance of DPP Based Polymers by Introducing One 3,4-Ethylenedioxythiophene Electron-Rich Building Block. *J. Mater. Chem. C* **2020**, *8*, 10859–10867. https://doi.org/10.1039/d0tc01047b.

(12) Zozoulenko, I.; Singh, A.; Singh, S. K.; Gueskine, V.; Crispin, X.; Berggren, M. Polarons, Bipolarons, and Absorption Spectroscopy of PEDOT. *ACS Appl. Polym. Mater.* **2019**, *1* (1), 83–94. https://doi.org/10.1021/acsapm.8b00061.

(13) Mitraka, E.; Jafari, M. J.; Vagin, M.; Liu, X.; Fahlman, M.; Ederth, T.; Berggren, M.; Jonsson, M. P.; Crispin, X. Oxygen-Induced Doping on Reduced PEDOT. *J Mater Chem A Mater* **2017**, *5* (9), 4404–4412. https://doi.org/10.1039/c6ta10521a.

(14) Ponder, J. F.; Gregory, S. A.; Atassi, A.; Menon, A. K.; Lang, A. W.; Savagian, L. R.; Reynolds, J. R.; Yee, S. K. Significant Enhancement of the Electrical Conductivity of Conjugated Polymers by Post-Processing Side Chain Removal. *J. Am. Chem. Soc.* **2022**, *144*, 1351–1360. https://doi.org/10.1021/jacs.1c11558.

(15) Wegner, B.; Lungwitz, D.; Mansour, A. E.; Tait, C. E.; Tanaka, N.; Zhai, T.; Duhm, S.; Forster, M.; Behrends, J.; Shoji, Y.; Opitz, A.; Scherf, U.; List-Kratochvil, E. J. W.; Fukushima, T.; Koch, N. An Organic Borate Salt with Superior P-Doping Capability for Organic Semiconductors. *Advanced Science* **2020**, *2001322*, 1–15. https://doi.org/10.1002/advs.202001322.

(16) Ratcliff, E. L.; Jenkins, J. L.; Nebesny, K.; Armstrong, N. R. Electrodeposited, “Textured” (e-P3HT) Films for Photovoltaic Applications Films for Photovoltaic Applications. *Chemical Materials* **2008**, *20* (18), 5796–5806.

(17) Heeg, J.; Kramer, C.; Wolter, M.; Michaelis, S.; Plieth, W.; Fischer, W. J. Polythiophene - O 3 Surface Reactions Studied by XPS. *Appl Surf Sci* **2001**, *180* (1–2), 36–41. https://doi.org/10.1016/S0169-4332(01)00316-6.

(18) Shen, D. E.; Lang, A. W.; Collier, G. S.; Anna, M. O.; Smith, E. M.; Tomlinson, L.; Reynolds, J. R. Enhancement of Photostability through Side Chain Tuning in Dioxythiophene-Based Conjugated Polymers. **2022**. https://doi.org/10.1021/acs.chemmater.1c03317.

(19) Hintz, H.; Peisert, H.; Egelhaaf, H. J.; Chasse, T. Reversible and Irreversible Light-Induced p-Doping of P3ht by Oxygen Studied by Photoelectron Spectroscopy (XPS/UPS). *Journal of Physical Chemistry C* **2011**, *115* (27), 13373–13376. https://doi.org/10.1021/jp2032737.

(20) Hüfner, S.; Wertheim, G. K.; Wernick, J. H. XPS Core Line Asymmetries in Metals. *Solid State Commun* **1975**, *17* (4), 417–422. https://doi.org/10.1016/0038-1098(75)90468-8.

(21) Easton, C. D.; Kinnear, C.; McArthur, S. L.; Gengenbach, T. R. Practical Guides for X-Ray Photoelectron Spectroscopy: Analysis of Polymers. *Journal of Vacuum Science & Technology A* **2020**, *38* (2), 023207. https://doi.org/10.1116/1.5140587.

(22) Harris, J. K.; Neelamraju, B.; Ratcliff, E. L. Intersystem Subpopulation Charge Transfer and Conformational Relaxation Preceding in Situ Conductivity in Electrochemically Doped Poly(3-Hexylthiophene) Electrodes. *Chemistry of Materials* **2019**, *31* (17), 6870–6879. https://doi.org/10.1021/acs.chemmater.9b01298.

(23) Major, G. H.; Fairley, N.; Sherwood, P. M. A.; Linford, M. R.; Terry, J.; Fernandez, V.; Artyushkova, K. Practical Guide for Curve Fitting in X-Ray Photoelectron Spectroscopy. *Journal of Vacuum Science & Technology A* **2020**, *38* (6), 061203. https://doi.org/10.1116/6.0000377.

(24) Bubnova, O.; Khan, Z. U.; Malti, A.; Braun, S.; Fahlman, M.; Berggren, M.; Crispin, X. Optimization of the Thermoelectric Figure of Merit in the Conducting Polymer Poly(3,4-Ethylenedioxythiophene). *Nat. Mater.* **2011**, *10*, 429–433. https://doi.org/10.1038/nmat3012.

(25) Zotti, G.; Zecchin, S.; Schiavon, G.; Louwet, F.; Groenendaal, L.; Crispin, X.; Osikowicz, W.; Salaneck, W.; Fahlman, M. Electrochemical and XPS Studies toward the Role of Monomeric and Polymeric Sulfonate Counterions in the Synthesis, Composition, and Properties of Poly(3,4-Ethylenedioxythiophene). *Macromolecules* **2003**, *36* (9), 3337–3344. https://doi.org/10.1021/ma021715k.

(26) Gregory, S. A.; Hanus, R.; Atassi, A.; Rinehart, J. M.; Wooding, J. P.; Menon, A. K.; Losego, M. D.; Snyder, G. J.; Yee, S. K. Quantifying Charge Carrier Localization in Chemically Doped

Semiconducting Polymers. *Nat. Mater.* **2021**, *20*, 1414–1421. https://doi.org/10.1038/s41563-021-01008-0.

(27) Snyder, G. J.; Snyder, A. H.; Wood, M.; Gurunathan, R.; Snyder, B. H.; Niu, C. Weighted Mobility. *Adv. Mater.* **2020**, *32*, 2001537. https://doi.org/10.1002/adma.202001537.

(28) Dongmin Kang, S.; Jeffrey Snyder, G. Charge-Transport Model for Conducting Polymers. *Nat Mater* **2017**, *16* (2), 252–257. https://doi.org/10.1038/nmat4784.
